# Biodiversity of Basidiomycetous Yeasts Associated with *Cladonia rei* Lichen in Japan, with a Description of *Microsporomyces cladoniophilus* sp. nov

**DOI:** 10.3390/jof9040473

**Published:** 2023-04-14

**Authors:** Ngoc-Hung Nguyen, Phuong-Thao Nguyen, Hitomi Otake, Ayana Nagata, Nobuharu Hirano, Yumi Imanishi-Shimizu, Kiminori Shimizu

**Affiliations:** 1 Department of Biological Science and Technology, Tokyo University of Science, Niijuku 6-3-1, Katsushika, Tokyo 125-8585, Japan; 2 College of Science and Engineering, Kanto Gakuin University, Mutsuura-higashi 1-50-1, Kanazawa-ku, Yokohama 236-8501, Kanagawa, Japan; 3 Medical Mycology Research Center, Chiba University, Inohana 1-8-1, Chuo-ku, Chiba 260-8673, Chiba, Japan

**Keywords:** basidiomycetes, *Cystobasidiomycetes*, meta-barcoding, *Microsporomycetaceae*, phylogeny, yeast cultures

## Abstract

For more than a century, lichens have been used as an example of dual-partner symbiosis. Recently, this has been challenged by the discovery of various basidiomycetous yeasts that coexist in multiple lichen species, among which *Cladonia* lichens from Europe and the United States were discovered to be highly specifically associated with the basidiomycetous yeast of the family *Microsporomycetaceae*. To verify this highly specific relationship, we investigated the diversity of basidiomycetous yeasts associated with *Cladonia rei*, a widely distributed lichen in Japan, by applying two approaches: yeast isolation from the lichen thalli and meta-barcoding analysis. We obtained 42 cultures of Cystobasidiomycetous yeast which were grouped into six lineages within the family *Microsporomycetaceae*. Unexpectedly, although the *cystobasidiomycetes*-specific primer was used, not only the cystobasidiomycetous yeasts but species from other classes were also detected via the meta-barcoding dataset; in particular, pucciniomycetous yeasts were found at a high frequency in some samples. Further, *Halobasidium xiangyangense*, which was detected in every sample with high abundance, is highly likely a generalist epiphytic fungus that has the ability to associate with *C. rei*. In the pucciniomycetous group, most of the detected species belong to the scale insect-associated yeast *Septobasidium* genus. In conclusion, even though *Microsporomyces* species are not the only yeast group associated with *Cladonia* lichen, our study demonstrated that the thalli of *Cladonia rei* lichen could be a suitable habit for them.

## 1. Introduction

Symbioses are widespread and essential to the diversity of our ecosystems. Lichen represents a successful model of the iconic symbiosis of stable dual mutualism of a single fungus and a photosynthesizing symbiont. With their long macro-evolutionary history, lichens can inhabit any environment or habitat and currently dominate nearly 10% of the Earth’s terrestrial ecosystem [1,2]. Among the lichen-forming fungi, *Lecanoromycetes*, as one of the largest clades, includes more than 80% of the lichen-forming species [3]. Belonging to this class, *Cladonia* is one of the most species-rich and morphologically distinctive genera of lichen-forming fungi, with a worldwide distribution [4].

Due to recent discoveries, lichens have been considered not only relevant to dual-partner symbiosis but also part of the complex ecosystems that are inhabited by other microscopic organisms, such as prokaryotes and fungi [5]. As lichens appeared 600 million years ago [6], the existence of secondary fungal species in Early Devonian (~415 million years ago) lichen fossils [7] indicates that this association might have existed throughout the evolutionary history of lichen. These coexistences have been detected in various lichen species [8,9,10]. As they have been mostly considered parasites or lichenicolous fungi [11], and a few as endolichenic fungi [12], and due to their rare isolation, the functions, influence, and roles on communities of lichens have been neglected or underestimated. The recent discovery by Spribille et al. [13] shed new light on this issue by proving the presence of basidiomycetous yeasts in the lichen cortex and their contribution to different phenotypes of two genetically inseparable *Bryoria* lichens. In the same study, the yeasts of the class *Cystobasidiomycetes* were detected in the cortex of various macro-lichen taxa, suggesting that the yeasts may represent the third essential constituent of lichen symbiosis. Multiple studies conducted to infer the diversity of lichen-associated basidiomycetous yeasts have delivered conflicting results. Although some supported the association between lichens and basidiomycetous yeasts [13,14,15], others argued against this association [16,17]. Spribille et al. [13], upon screening the lichen-associated yeasts from a wide range of lichen species, detected a specific group of basidiomycetous yeasts, *Cyphobasidiales* (*Pucciniomycotina*, *Basidiomycota*), associated with multiple species, but the yeasts detected from *Cladonia* lichens were grouped into one lineage within the *Microsporomycetaceae* family (*Cystobasidiomycetes*, *Pucciniomycotina*, *Basidiomycota*). Later, these unknown yeasts were discovered in a widespread species of this genus, despite their reproductive strategies [14,18]. This showed the high specificity of association with basidiomycetous yeasts of *Cladonia* lichen, while the reproductive and dispersal strategies were shown to be the key factors shaping photobiont diversity in this lichen group [18]. Surveys of these *Cladonia*-associated yeasts have been lacking in most of Asia, where several species from the same family have been discovered recently [19].

Most studies that have attempted to discover the biodiversity of lichen-associated basidiomycetous yeasts have utilized one or two approaches that might lead to bias. In some studies, Sanger sequencing, which provides high confidence for detected taxa from sequences, was utilized to determine the lichen-associated yeasts [16], but this approach is not sensitive enough for low abundance symbionts, and the probability of detection of certain species significantly depends on the biodiversity of the community within the lichens [20]. Although isolation from the thallus fragments method seems to have provided decent evidence of the existence of lichen-associated yeasts, it is highly limited by such factors as the type of culture media used for isolation and the skills of technicians [21,22]. Further, the contamination rate of cultures from *Cladonia* lichens due to their natural habitat [22] increases the difficulty of revealing the true diversity of the yeast community within the thalli of these lichens. Given advances in sequencing technologies, the meta-barcoding approach with new emerging high-throughput sequencing techniques seems to be a powerful tool for detecting the true diversity of the basidiomycetous yeast community within lichen thalli [23,24,25,26]. However, the meta-barcoding method also suffers numerous biases, so it requires affirmation from other methods [27,28,29].

Here, we focused on the biodiversity of basidiomycetous yeasts associated with *Cladonia rei*, which is widely distributed in Japan, using multiple approaches to infer the existence of lichen-associated yeast species and determine whether there is a high specificity in the third symbionts of *Cladonia* lichen in the region. The yeasts were isolated from multiple lichen specimens collected from various locations. The basidiomycetous yeasts were further screened via meta-barcoding analysis of conserved genomic sequences, amplified with the basidiomycete-specific primers designed by Spribille et al. [13].

## 2. Materials and Methods

### 2.1. Sampling

Fifteen specimens of *Cladonia rei* thallus were collected from two locations in Chiba prefecture, Japan, from March 2017 to November 2018 (Table 1). The species was confirmed via morphological and molecular methods. The collected thallus was frozen and stored at −20 °C.

### 2.2. Isolation, Culturing, and Characterization of the Yeast Strains

Strains were isolated from the thallus using the method described by Yamamoto et al. [22]. The thalli were inspected for external signs of discoloration and parasitism. For each isolation, around 10 podetia were selected and washed with running water for at least 1 h and then with 7 mL 1% sodium hypochlorite. The washed podetia were rinsed with sterilized distilled water thoroughly several times and then ground into small pieces using a mortar and pestle. Finally, the thallus fragments with sizes between 150 and 500 µm were selected using two nylon sieve meshes and then placed onto Malt/Yeast extract (YM) medium [30].

Yeasts grown from the thallus fragments were isolated into axenic cultures and were then maintained on YM medium at 25 °C. Morphological, physiological, and biochemical characteristics were examined following the methods of Kurtzman et al. [31]. We tested for ballistoconidia using the inverted plate method [32]. The glass slide containing the discharged spores was removed for examination under the microscope after 3–10 days. The potential sexual cycles were investigated using the Dalmau plate method on corn meal agar (CMA), YM agar (YMA), and potato–dextrose agar (PDA), according to Kurtzman et al. [31]. A loopful of cells of each strain was streaked on an agar plate and incubated at 17 °C for 1 month, and the cultures were examined microscopically every 2 weeks.

### 2.3. Sequencing and Phylogenetic Analyses

The total DNA of *C. rei* thallus and the yeast cultures was extracted by the method of Nakada et al. [33]. The ITS rDNA sequences, which were amplified with ITS4 and ITS5 primer, were used to confirm the species of lichen thallus [34]. For the isolated yeasts, the ITS region and partial LSU rDNA were amplified with ITS4 and ITS5; LR0 and LR5 primers were used, respectively [34,35]. PCR amplification for ITS began with denaturation at 95 °C for 5 min, followed by 35 cycles of denaturation at 95 °C for 30 s, annealing at 55 °C for 1 min, and elongation at 72 °C for 1 min, finished with extension at 72 °C for 5 min. PCR amplification was carried out for LSU denaturation at 95 °C for 5 min, which was followed by 35 cycles of denaturation at 95 °C for 30 s, annealing at 55 °C for 1 min, and elongation at 68 °C for 2 min, and finished with extension at 72 °C for 5 min. The PCR products were purified using a FastGene^TM^ Gel/PCR extract kit (Nippon Genetics, Tokyo, Japan). The forward and reverse strands of the purified PCR products were amplified using the same primers with a BigDye^TM^ Terminator v3.1 Cycle Sequencing Kit (Applied Biosystems, Waltham, MA, USA), following purification with Sephadex G-50. Sanger sequencing was conducted with a 3130xl Genetic Analyzer and a 3500 Genetic Analyzer (Applied Biosystems). The final sequences were assembled using Genetyx v. 13 (GENETYX, Tokyo, Japan).

To infer the phylogenetic position of our strains within the class *Cystobasidiomycetes*, we conducted a phylogenetic analysis on the basis of the phylogenetic results from Li et al. [19] and Černajová and Škaloud [14]. Sequences of the ITS region and LSU rDNA of species in the *Cystobasidiomycetes* class retrieved from GenBank, together with the sequences of the obtained cultures (Table S1), were aligned using MAFFT using the Q-INS-I method; each region was aligned separately. Gblocks v. 0.91b was used to remove the ambiguously aligned region [36]. Substitution models were estimated with Akaike Information Criterion (AIC) using JModelTest v.2.1.4 [37]. The model GTR+ I + G was selected for both LSU and ITS. Our cultures all clustered within the family *Microsporomycetaceae* (see Results). Thus, we reconstructed the phylogeny of this family with the currently accepted species [19] and our cultures. *Bannoa bischofiae* and three *Erythobasidium* species (*Erythrobasidiaceae*, *Cystobasidiomycetes*, *Pucciniomycotina*, *Basidiomycota*) were selected as outgroups. The sequences were processed as described above. We used RAxML-HPC v.8 to construct the maximum likelihood (ML) phylogeny [38] and perform 1000 rapid bootstrap replicates. The output trees were visualized using FigTree v.1.4.4 [39]. The final presentation was undertaken in free RStudio software [40,41] with the packages ggtree [42] and treeio [43].

### 2.4. Meta-Barcoding Sequencing and OTU Identity Analysis

Seven specimens were selected for meta-barcoding (Table 1). The yeast ITS rDNA from the DNA of C. rei was amplified using the *Cystobasidiomycetes*-specific primer ITS_symrho_2F and LR0_symrho_R designed by Spribille et al. [13]. PCR products were checked on 2% agarose gel and quantified using Qubit 2.0. The libraries were generated with NEBNext^®^Ultra™ DNA Library Prep Kit for Illumina^®^ (San Diego, CA, USA). Sequencing was completed with the paired-end protocol 2 × 300 bp on a PE300 MiSeq instrument at Novogene, Tokyo, Japan. All output reads are available in the Short Read Archive under accession PRJNA765160. Raw paired-end reads were merged using FLASH v.1.2.7 [44]. The adapters and barcodes were trimmed from sequences using fastx_trimmer, implemented in FASTX-Toolkit [45]. The operational taxonomic units (OTUs) were identified by clustering the trimmed sequences based on the sequence similarity threshold (97%) using USEARCH v.11 [46]. Each OTU’s taxonomy assignment was performed by blasting their reference sequences [47] against the full “UNITE + INSD” dataset for fungi version 2021-05-10 [48] with a 10 × 10^−50^ e-value threshold. The OTUs that were identified as cystobasidiomycetous yeasts and showed a relative abundance in any sample higher than 1% were further included in the phylogenetic analysis, with ITS region data to determine approximately their position within the class using the same method as above. To visualize the yeast community composition, we used the free RStudio software [40,41] with the ggplot2 package [49]. To avoid an over-detailed graph, the OTUs with a relative abundance of less than 1% were grouped.

## 3. Results

### 3.1. Isolated Cultures

**Figure 1:**
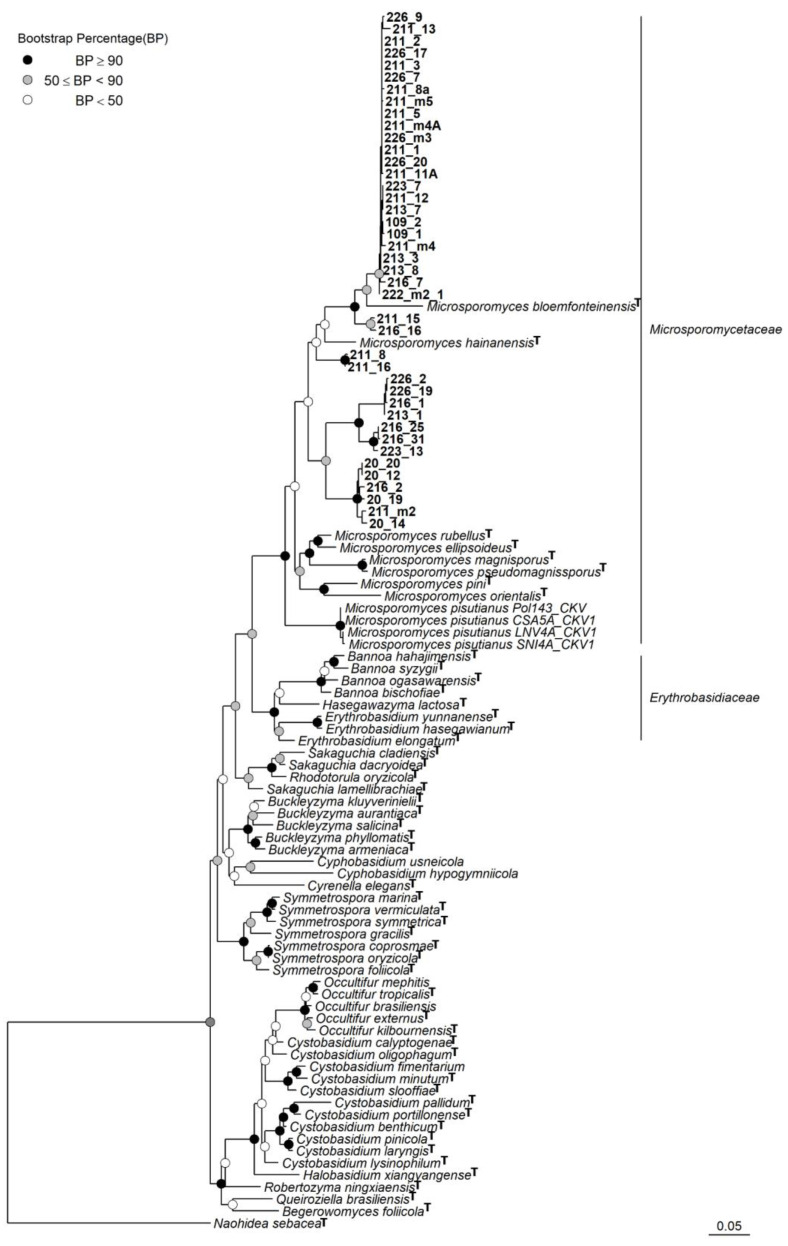
Phylogenetic tree of *Cystobasidiomycetes* obtained via the maximum likelihood analysis of the combined sequences of the LSU rDNA and ITS regions. **^T^** indicates the sequence from the holotype strain was used.

**Figure 2:**
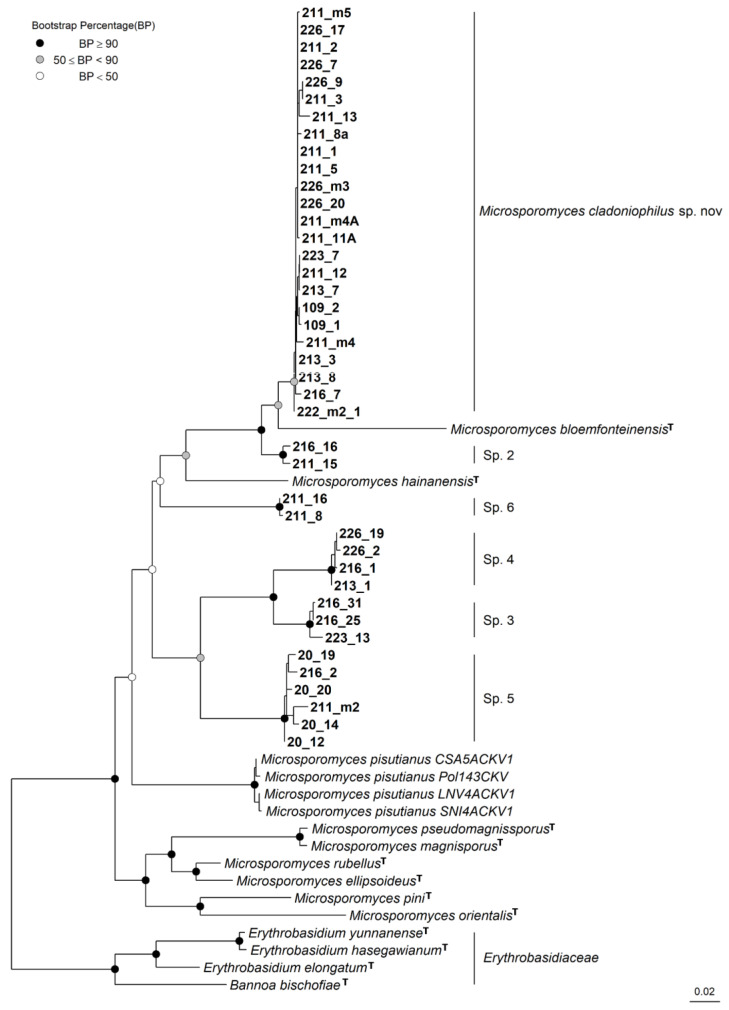
Phylogenetic tree of *Microsporomycetaceae* obtained by maximum likelihood analysis of the combined sequences of the LSU rDNA and ITS regions. **^T^** indicates the sequence from the holotype strain was used.

Forty-two strains were cultured successfully from eight *C. rei* specimens and identified by sequencing the ITS and LSU rDNA (Table 2). Our phylogenetic analyses showed a consistent topology and were generally congruent with most of the major groups from [19] (Figure 1). Furthermore, the phylogenetic analyses also grouped *Lichenozyma* species with *Microsporomyces* species and our cultures. Hence, we agree with Li et al. (2020) that the *Lichenozyma* genus should be considered synonymous with *Microsporomyces*. Although the phylogenetic analysis of the *Cystobasidiomycetes* class was slightly incongruent in topology with the analysis on the family *Microsporomycetaceae*, both indicated that all of the isolated cultures belonged to *Microsporomycetaceae*, with high statistical support. Furthermore, the phylogenetic analysis of *Microsporomycetaceae* showed that our cultures could be grouped into six distinct lineages (Figure 2). The first lineage (*Microsporomyces cladoniophilus*) comprised most of the strains (24 strains) that appeared to be a sister of *Microsporomyces bloemfonteinensis* and the second lineage (Sp.2), with two isolated cultures 211_15 and 216_16 as their sister, with high support. The rest of the cultures were divided into two sister pairs, in which one was a sister of *Microsporomyces hainanensis*, but their relationships were not well supported. Furthermore, our phylogenetic results were congruent with Li et al. [19] in terms of the placement of the newly described *Lichenozyma* genus [14] (*Microsporomyces pisutianus* in Figure 1 and Figure 2) inside the *Microsporomyces* genus, with high statistical support, so we proposed *Lichenozyma* as a synonym of *Microsporomyces*.

### 3.2. Meta-Barcoding Results

**Figure 3:**
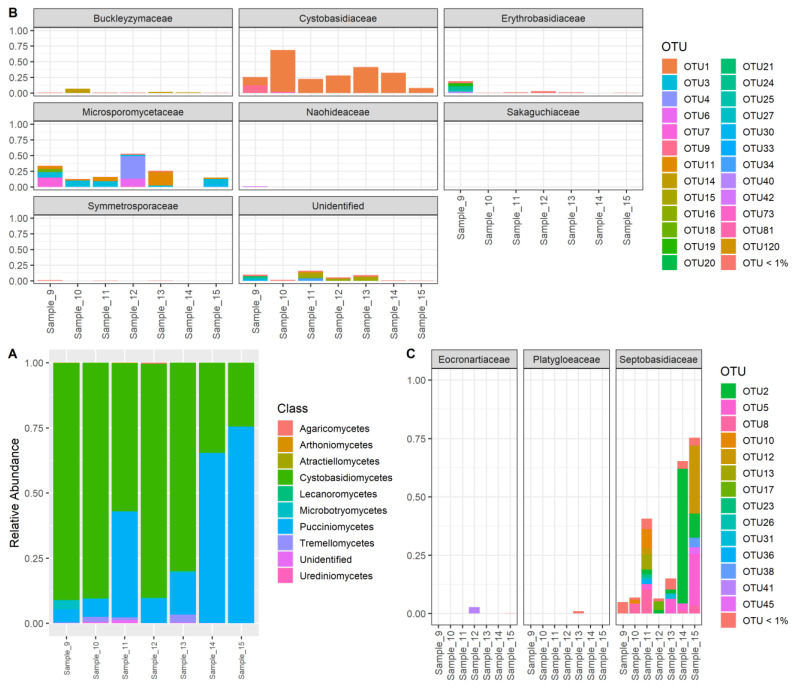
The Proportion of OTUs in meta-barcoding reads. (**A**) Relative abundance at the class level; (**B**) OTU level corresponding to each family within the *Cystobasidiomycetes* class; (**C**) OTU level corresponding to each family within the *Pucciniomycetes* class. The OTUs with low abundance (<1%) were grouped together.

A total of 3,809,740 reads were generated and clustered into 348 OTUs that mostly belonged to the *Cystobasidiomycetes* and *Pucciniomycetes* classes (Figure 3A). Within the *Cystobasidiomycetes* class, most of the OTUs with high relative abundance belonged to the two families *Cystobasidiaceae* and *Microsporomycetaceae* (Figure 3B). Based on the relative abundance of meta-barcoding reads, the OTU1, which occurred most abundantly in every sample, belonged to the *Cystobasidiaceae* family, along with two other OTUs (OTU9 and OTU81) (Figure 3B). We detected a high diversity of *Microsporomycetaceae* from our samples (six out of seven samples) since various OTUs were classified as belonging to the *Microsporomycetaceae* family. There were only three families from the *Pucciniomycetes* class detected from our data, and most of the OTUs were classified as *Septobasidiaceae* (Figure 3C). To infer the detailed phylogenetic positions of these OTUs, we conducted phylogenetic analyses on the OTUs that were identified as *Cystobasidiomycetes* and *Pucciniomycetes* fungi using the same method as described above. From the phylogenetic tree (Figure S1), OTU 14, 40, 9, and 1 were identified as *Buckleyzyma aurantiaca*, *Erythrobasidium hasegawianum*, *Occultifur mephitis*, and *Halobasidium xiangyangense*. This was consistent with the results from blasting against the “UNITE + INSD” dataset. Further, thirteen OTUs were grouped with the *Microsporomyces* species or our cultures, which suggested they belonged to these genera, while the remaining eight OTUs (19, 20, 21, 24, 25, 27, 33, 42) were unable to be classified. For the OTUs that belonged to the *Pucciniomycetes* class, most were identified as *Septobasidum* species, which are known for their association with scale insects (Figure S2), except OTU 41, which was classified as *Eocronartium* species (Figure 3C).

### 3.3. Taxonomy

**Figure 4:**
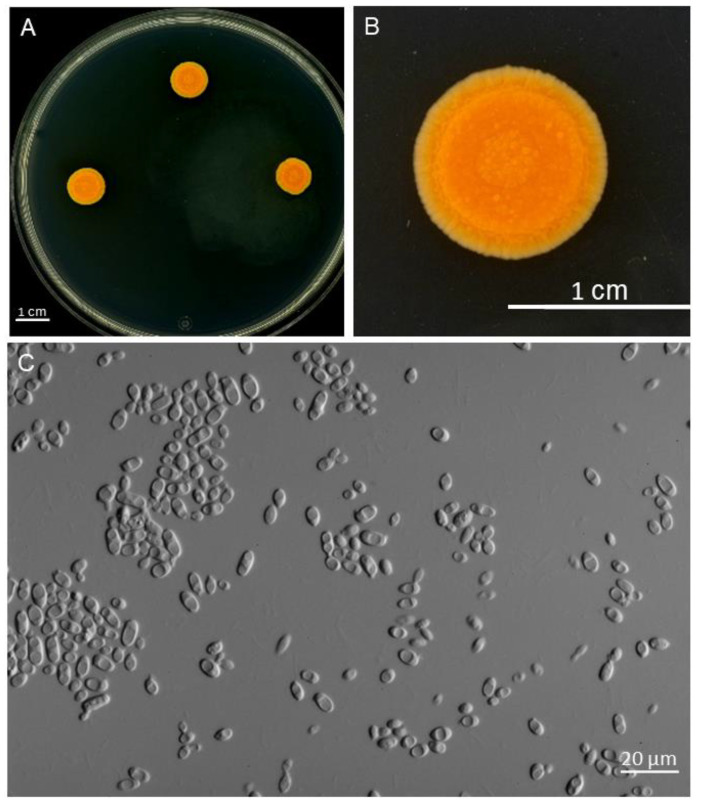
Colonies (**A**,**B**) and cells (**C**) of *Microsporomyces cladoniophilus* sp. nov on MYA after 10 days.

(1) *Microsporomyces cladoniophilus* N.H. Nguyen, P.T. Nguyen, Otake, Ayana Nagata, Imanishi and K. Shimizu sp. nov. (Figure 4).

MycoBank Number: MB846812.

With the evidence from both the culture of thallus fragments and DNA meta-barcoding, we suggest a novel *Microsporomyces* species which is associated with *C. rei* lichen.

Holotype: The strain 211_1 was isolated by H. Otake from the thallus of *Cladonia rei* collected in Chiba, Japan (N35.18731, E140.11192) in October 2017. It was chosen as the holotype. This culture is permanently preserved in a metabolically inactive state under the number NBRC 115437 and CBS 17989. Strains 109_1 and 226_20 were selected as the paratype. These cultures are permanently preserved in a metabolically inactive state under the number NBRC 115348 and NBRC 115439, respectively.

*Diagnosis:* Phylogenetic analysis indicated the closest species of *M. cladoniophilus* was *M. bloemfonteinensis*. *M. cladoniophilus* differed from *M. bloemfonteinensis* by 22–25 nucleotides (~5%) and 33–36 nucleotides (~10%) mismatched in the LSU and ITS regions, respectively. Physiologically, *M. cladoniophilus* differs from its closely related species, *M. bloemfonteninensis*, in its ability to assimilate sucrose, glycerol, D + galactose, potassium nitrate, and L-lysine, and its inability to assimilate melibiose, D-melezitose, inulin, D-ribose, DL-lactic acid, succinic acid salt, and inositol, as well as its inability to grow in vitamin-free medium, and its ability to produce urease.

**Host range and distribution:** Currently only reported from *Cladonia rei* lichen from Chiba Prefecture, Japan.

**Ecology:** Endolichenic fungus associated with *Cladonia rei* (*Cladoniaceae*, *Lecanoromycetes*) and lichen; no signs of damage were observed.

**Culture characteristics:** On YMA, after 10 days at 25 °C, colonies were around 1 cm in diameter, the surface was smooth or rough, orange to dark orange colored, depressed in the center, and the margin was undulating or entire (Figure 4A,B). The cells were ovoid and ellipsoidal, 2.1–5.4 µm × 3.0–10.3 µm (measured from 10 random cells, length/width ratio 1.45–2.54), and single, and budding was polar (Figure 4C). In Dalmau plate culture on YM, CM, PDA, and YMA, pseudohyphae were not formed, and sexual structures were not observed. Ballistoconidia were not produced.

**Physiological and biochemical characteristics:** Glucose fermentation ability was negative. Glucose, L-Sorbose (weak or slow), Sucrose, Maltose, D + Cellobose, D + trehalose, Lactose (positive or weak or latent), Raffinose (weak or latent), D + Xylose, Glycerol, D-Mannitol, D-Glucitol, Gluconic acid, D-Glucuronic acid, Salicin (positive or weak), D + Galacturonic acid, D + Glucono-1,5-lactone, Xylitol, Ribitol, D + Galactose, Ethanol (weak), and N-Acetyl-D + Glucosamine (positive or weak or negative) were assimilated as sole carbon sources. Melibiose, D-Melezitose, Inulin, Starch soluble, L + Arabinose, D-Arabinose, D-Ribose, α-L + Rhamnose, D-Glucosamine, meso-Erythritol, α-methyl-D + Glucoside, DL-Lactic acid, Succinic acid, Citric acid, Inositol, 2,3-Butanediol, L-Arabitol, Galactitol, 1,2-Propanediol, Hexadecane, D + Galactose, and 2-keto-D-Gluconate were not assimilated. Nitrate, nitrite (weak), L-lysine, and cadaverine (weak) were utilized as nitrogen sources, while Ethylamine was not utilized. No starch-like substances were produced. Growth on 50% (*w*/*w*) glucose-yeast extract agar was negative. Diazonium Blue B reaction was positive. Urease activity was positive. Growth in a vitamin-free medium was negative (Table 3).

(2) *Microsporomyces pisutianus* (Černajová and Škaloud) N.H. Nguyen, P.T. Nguyen, Imanishi and K. Shimizu, comb. nov.

= *Lichenozyma pisutiana* Černajová and Škaloud.

MycoBank Number: MB847500.

*Lichenozyma pisutianus* Černajová and Škaloud, which is located in the *Microsporomyces* clade (*Microsporomyces pisutianus* in Figure 1, Figure 2 and Figure S1), was proposed as a new combination in *Microsporomyces*.

## 4. Discussion

With a focus on investigating the diversity of the lichen-associated *Cystobasidiomycete* yeasts in *C. rei* lichen, we discovered multiple species belonging to this class from the thalli of this lichen. With multiple isolated strains, a new *Cystobasidiomycete* species inhabiting the thallus of *C. rei* was discovered and confirmed by meta-barcoding analysis.

The high level of specificity of *Cystobasidiomycetes* yeasts was speculated by previous studies [13,14,52]. For instance, it was thought the *Microsporomycetaceae* family might be specifically associated with the *Cladonia* lichens and *Cystobasidiales* with the lichens of the *Parmeliaceae* family. Our results were in agreement with the specific partnership of *Cladonia* lichen, but the relationship is not strictly specialized. Alongside *Microsporomyces cladoniophilus* sp. nov., which was detected in most of the samples, we further isolated five unknown species that belong to the *Microsporomyces* genus. These species were also detected by the meta-barcoding analyses, which confirmed the diversity of this yeast genus within the thallus of *Cladonia* lichen [15,16]. Despite the new yeast species not being isolated from the meta-barcode sequenced specimens, the sequences of these species were still detected in most of the samples. As the meta-barcoding specimens were collected in early winter, whereas the others were collected in early summer, the difference in climate may have contributed to this result [53,54].

From the meta-barcoding reads, we found that another *Cystobasidiomycete* yeast, *Halobasidium xiangyangense*, existed in every specimen, with the highest frequency of reads among the other OTUs and our isolated strains. While *Halobasidium xiangyangense* was first described from an isolated culture taken from pickling sauce [55], this species is likely an epiphyte fungus, as it has since been isolated from plant materials worldwide [56,57,58,59]. As generalist species are usually not constrained by the environment and can occupy a wide range of habitats [60,61], this epiphyte fungus is highly likely to be a generalist with the ability to associate with *Cladonia* lichen. Unexpectedly, at least one *Septobasidium* species, a scale insect-associated fungi [62,63], was detected in each specimen. There have not been many studies on this fungal group, but their existence in the thalli of lichen might indicate that they are also a generalist species.

Wang et al. [64] first described the family *Microsporomycetaceae* on the basis of the molecular data of five species. To accommodate the yeasts that have been reported as inhabitants of *Cladonia* lichens in Europe and the United States [13,14], the genus *Lichenozyma* has been proposed. Based on multiple loci phylogeny, Li et al. [19] objected to this proposal and suggested this genus is a synonym for *Microsporomyces*. Our phylogenetic results agreed with theirs and further indicated that the diversity of the genus *Microsporomyces* remains underestimated, with several unknown isolated strains grouped in this genus.

In our study, the effort to directly detect *Cystobasidiomycete* yeasts from the specimens with the *Cystobasidiomycetes*-specific primer was not successful; this was probably influenced by the biodiversity of the co-existing cystobasidiomycetous yeasts within the thallus [20]. Our results from the meta-barcoding analysis showed more than two *Cystobasidiomycete* species associated with *C. rei* lichen, and neither of them had an absolute abundance (>70% of total reads) in all samples determined. In conclusion, our study re-confirmed the existence of yeast from the *Microsporomycetaceae* family within the thalli of *Cladonia rei* lichen, and the detected *H. xiangyangense* further highlighted the diversity and complexity of the yeast community associated with this lichen.

## Figures and Tables

**Table 1:** Information of the collected specimens of *Cladonia rei*. “X” indicates the specimens used in the methods.

Specimen ID	Lichen Species	Locality	Collecting Date	Lichen Thalli Isolating	Meta-Barcoding	Herbarium ID
1	*Cladonia rei*	Kimitsu, Chiba N35.18731, E140.11192	Mar. 2017	X		CBM-FL-203638
2	*Cladonia rei*	Odawara, Kanagawa N35.24410, E139.11775	Oct. 2017	X		CBM-FL-206689
3	*Cladonia rei*	Ichihara, Chiba N35.30606, E140.12924	Jun. 2018	X		CBM-FL-103149
4	*Cladonia rei*	Ichihara, Chiba N35.30591, E140.12884	Jun. 2018	X		CBM-FL-103150
5	*Cladonia rei*	Kimitsu, Chiba N35.19563, E140.07037	Jun. 2018	X		CBM-FL-103152
6	*Cladonia rei*	Kimitsu, Chiba N35.19563, E140.07037	Jun. 2018	X		CBM-FL-103153
7	*Cladonia rei*	Kimitsu, Chiba N35.20902, E140.09221	Jun. 2018	X		CBM-FL-103154
8	*Cladonia rei*	Kimitsu, Chiba N35.20820, E140.09119	Jun. 2018	X		CBM-FL-103155
9	*Cladonia rei*	Ichihara, Chiba N35.305886, E140.12929	Nov. 2018	X	X	CBM-FL-205144
10	*Cladonia rei*	Ichihara, Chiba N35.305886, E140.12929	Nov. 2018	X	X	CBM-FL-205145
11	*Cladonia rei*	Ichihara, Chiba N35.305886, E140.12929	Nov. 2018	X	X	CBM-FL-205146
12	*Cladonia rei*	Ichihara, Chiba N35.305858, E140.128931	Nov. 2018	X	X	CBM-FL-205147
13	*Cladonia rei*	Kimitsu, Chiba N35.195252, E140.07036	Nov. 2018	X	X	CBM-FL-205148
14	*Cladonia rei*	Kimitsu, Chiba N35.19493, E140.071076	Nov. 2018	X	X	CBM-FL-205149
15	*Cladonia rei*	Kimitsu, Chiba N35.19493, E140.071076	Nov. 2018	X	X	CBM-FL-205150

**Table 2:** List of the sequences from obtained cultures and their Genbank accession numbers.

Lineage	Strain/Voucher	ITS	LSU	Specimen ID
*Microsporomyces cladoniophilus* sp. nov	109_1	MZ505462	MZ513978	2
*Microsporomyces cladoniophilus* sp. nov	109_2	MZ505463	MZ513979	2
*Microsporomyces cladoniophilus* sp. nov	211_1	MZ505473	MZ513980	1
*Microsporomyces cladoniophilus* sp. nov	211_11A	MZ505468	MZ513983	1
*Microsporomyces cladoniophilus* sp. nov	211_12	MZ505469	MZ513984	1
*Microsporomyces cladoniophilus* sp. nov	211_13	MZ505470	MZ513985	1
*Microsporomyces cladoniophilus* sp. nov	211_2	MZ505474	–	3
*Microsporomyces cladoniophilus* sp. nov	211_3	MZ505475	–	3
*Microsporomyces cladoniophilus* sp. nov	211_5	MZ505476	MZ513981	3
*Microsporomyces cladoniophilus* sp. nov	211_m5	MZ505482	MZ513990	3
*Microsporomyces cladoniophilus* sp. nov	211_8a	MZ505477	MZ513982	3
*Microsporomyces cladoniophilus* sp. nov	211_m4	MZ505481	MZ513988	3
*Microsporomyces cladoniophilus* sp. nov	211_m4A	MZ505480	MZ513989	3
*Microsporomyces cladoniophilus* sp. nov	213_3	MZ505484	MZ513992	3
*Microsporomyces cladoniophilus* sp. nov	213_7	MZ505485	MZ513993	3
*Microsporomyces cladoniophilus* sp. nov	213_8	MZ505486	MZ513994	3
*Microsporomyces cladoniophilus* sp. nov	216_7	MZ505492	–	3
*Microsporomyces cladoniophilus* sp. nov	222_m2_1	MZ505493	MZ514000	3
*Microsporomyces cladoniophilus* sp. nov	223_7	MZ505495	MZ514001	3
*Microsporomyces cladoniophilus* sp. nov	226_17	MZ505496	–	3
*Microsporomyces cladoniophilus* sp. nov	226_20	MZ505498	MZ514006	3
*Microsporomyces cladoniophilus* sp. nov	226_7	MZ505500	–	4
*Microsporomyces cladoniophilus* sp. nov	226_9	MZ505501	MZ514004	4
*Microsporomyces cladoniophilus* sp. nov	226_m3	MZ505502	MZ514007	4
*Microsporomyces* Sp.2	211_15	MZ505471	MZ513986	6
*Microsporomyces* Sp.2	216_16	MZ505487	MZ513997	13
*Microsporomyces* Sp.6	211_16	MZ505472	–	6
*Microsporomyces* Sp.6	211_8	MZ505478	–	6
*Microsporomyces* Sp.5	20_12	MZ505464	MZ513974	4
*Microsporomyces* Sp.5	20_14	MZ505465	MZ513975	6
*Microsporomyces* Sp.5	20_19	MZ505466	MZ513976	6
*Microsporomyces* Sp.5	20_20	MZ505467	MZ513977	6
*Microsporomyces* Sp.5	211_m2	MZ505479	MZ513987	14
*Microsporomyces* Sp.5	216_2	MZ505490	MZ513996	13
*Microsporomyces* Sp.3	216_25	MZ505489	MZ513998	13
*Microsporomyces* Sp.3	216_31	MZ505491	MZ513999	13
*Microsporomyces* Sp.3	223_13	MZ505494	MZ514002	13
*Microsporomyces* Sp.4	213_1	MZ505483	MZ513991	10
*Microsporomyces* Sp.4	216_1	MZ505488	MZ513995	10
*Microsporomyces* Sp.4	226_19	MZ505497	MZ514005	13
*Microsporomyces* Sp.4	226_2	MZ505499	MZ514003	13

**Table 3:** Physiological and biochemical characteristics of *Microsporomyces cladoniophilus* sp. nov. and other species in the *Microsporomycetaceae* family.

	*Microsporomyces cladoniophilus* sp. nov.	*Microsporomyces magnisporus* ^a^	*Microsporomyces pseudomagnisporus* ^d^	*Microsporomyces rubellus* ^d^	*Microsporomyces ellipsoideus* ^d^	*Microsporomyces pini* ^b^	*Microspomyces bloemfonteinensis* ^b^	*Microsporomyces orientalis* ^b^	*Microsporomyces hainanensis* ^c^
D + Glucose	+	+	+	+	+	+	+	+	+
L-Sorbose	w/s	+(w)	+	+	+	−	−	−	n/a
Sucrose	+	+(−)	+	−	−	−	−	+	n/a
Maltose	+	+(w)	+	+	+	−	+	+	−
D + Cellobose	+	−	−	+	+	−	+	+	+
D + Trehalose	+	+	−	−	−	−	+	+	+
Lactose	+/w/l	−	w	+	+	+	+	+	−
Melibiose	−	+	−	−	−	−	+	+	n/a
Raffinose	w/l	+(w)	w	+	w	−	+	+	n/a
D-Melezitose	−	+(w)	w	+	+	−	+	w	−
Inulin	−	+	w	+	−	w	+	+	−
Starch soluble	−	+(w)	l	−	−	−	−	−	+
D + Xylose	+	+(s)/−	−	−	+	−	+	w	w
L + Arabinose	−	+(w)/−	−	−	−	−	−	+	−
D-Arabinose	−	−	−	−	−	−	−	+	n/a
D_Ribose	−	+(s)/−	w	−	−	−	+	+	+
α_L + Rhamnose	−	+(w)/−	−	−	−	−	w	+	n/a
D-Glucosamine	−	+(l/w)/−	−	−	−	−	−	−	n/a
Glycerol	+	+/−	−	−	−	−	−	−	n/a
meso_Erythritol	−	−	−	−	−	+	−	+	−
D-Mannitol	+	+(w)	w	lw	−	+	w	w	+
D_Glucitol	+	+(w)	w	w	+	−	w	+	−
α methyl-D + Glucoside	−	+(w)/−	w	−	+	−	−	+	n/a
Gluconic acid	+	+	n/a	n/a	n/a	+	+	+	n/a
DL Lactic acid	−	+(w)	−	v	−	+	+	+	−
Succinic acid salt	−	+	w	−	lw	+	+	+	−
Citric acid	−	+(l)	−	−	−	+	−	+	−
D-Glucuronic acid	+	+	n/a	n/a	n/a	n/a	n/a	n/a	n/a
Inositol	−	−	−	−	−	−	w	−	−
Salicin	+/w	+(w)/−	−	v	+	+	+	+	−
D + Galacturonic acid	+	+(s)	n/a	n/a	n/a	n/a	n/a	n/a	n/a
2,3-Butanediol	−	+(l/w)/−	n/a	n/a	n/a	n/a	n/a	n/a	n/a
D + Glucono-1,5-lactone	+	+	n/a	n/a	n/a	n/a	n/a	n/a	n/a
Xylitol	+	+(w)	n/a	n/a	n/a	n/a	n/a	n/a	n/a
Ribitol	+	+(w)	w	+	−	−	+	−	n/a
L-Arabitol	−	−	n/a	n/a	n/a	n/a	n/a	n/a	n/a
Galactitol	−	−	−	+	−	−	−	−	n/a
1,2-Propanediol	−	−	n/a	n/a	n/a	n/a	n/a	n/a	n/a
Hexadecane	−	n/a	−	−	−	−	−	−	−
D + Galactose	+	+(w)	+	+	+	−	−	−	n/a
Ethanol	w	−	+	−	−	−	w	+	−
Methanol	−	−	−	−	−	−	−	−	−
N-Acetyl-D(+)Glucosamine	+/w/−	+	−	n/a	n/a	+	−	+	n/a
2-keto-D-gluconate (2-Oxoglutaric acid)	−	+	n/a	n/a	n/a	n/a	n/a	n/a	n/a
Cadaverine	w	−	+	−	−	+	n/a	+	n/a
Potassium nitrate	+	+	+	v	+	−	−	+	+
Sodium nitrite	w	+	−	−	−	−	+	+	+
Ethylamine	−	−	+	−	−	−	−	+	−
L-Lysine	+	−	+	v	w	+	−	+	−
Fermentation of glucose	−	−	−	−	−	−	−	−	−
Growth in vitamin-free medium	−	−	+	−	−	−	+	+	n/a
Growth at 22 °C	+	+	−	+	+	n/a	n/a	n/a	n/a
Growth at 25 °C	+	+	−	+	+	+	+	+	+
Growth at 30 °C	−	+	−	+	−	n/a	n/a	n/a	n/a
Growth at 32 °C	−	−	−	w	−	n/a	n/a	n/a	n/a
Growth at 35 °C	−	−	−	−	−	n/a	n/a	n/a	−
Growth with 50% glucose	−	−	−	−	−	−	−	−	n/a
Starch like compounds formation	−	−	−	−	−	−	−	−	−
Hydrolysis of Urea	+	+	+	+	+	+	−	−	n/a
Diazonium blue B reaction	+	+	+	+	+	n/a	n/a	n/a	+

Abbreviations: +, positive; −, negative; l, latent; w, weak; lw, latent and weak; v, variable; n/a, not available. ^a^ Results obtained from Nakase et al. [50]; ^b^ Results obtained from Pohl et al. [51]; ^c^ Results obtained from Bai et al. [52]; ^d^ Results obtained from Li et al. [19].

## Data Availability

The *Microsporomyces cladoniophilus* sp. nov holotype (211_1) is available in the NBRC (no. 115437) and CBS (no. 17989) strain collections. The paratypes (109_1 and 226_20) are available in the NBRC (no. 115348 and no. 115349) strain collection. The nucleotide sequences are available in GenBank (Table S1). The meta-barcoding raw reads were deposited on the Sequence Read Archive (SRA) data in NCBI under accession number SRR16004934.

## Supplementary Materials

The following supporting information can be downloaded at: https://www.mdpi.com/article/10.3390/jof9040473/s1, Table S1: List of sequences retrieved from GenBank used for phylogenetic analyses; Table S2: The OTUs list with taxonomic identification and their read counts in each sample.; Figure S1. Phylogeny tree of *Cystobasidiomycetes* obtained via the maximum likelihood analysis of the combined sequences of the LSU rDNA and ITS regions. The OTUs from the meta-barcoding sequences were marked with red; Figure S2 Phylogeny tree of *Septobasidium* obtained by maximum likelihood analysis of the ITS sequences. The OTUs from the meta-barcoding sequences were marked with red color.
