# Supplementary material for: Biodiversity of Basidiomycetous Yeasts Associated with Cladonia rei Lichen in Japan, with a Description of Microsporomyces cladoniophilus sp. nov
Source: J Fungi (Basel). 2023 Apr 14;9(4):473. doi: 10.3390/jof9040473 (PMC10145395; doi:10.3390/jof9040473)
Supplement: Supplementary file 1 [file jof-09-00473-s001.zip › jof-2157093-supplementary.pdf]

Table S1. List of sequences retrieved from GenBank used for phylogenetic analyses. "TYPE" indicates the holotype strain.

Taxon	Strain/voucher	ITS	LSU
Bannoa bischofiae	JCM 10338 (TYPE)	AB035721	AB082572
Bannoa hahjimensis	JCM 10336 (TYPE)	AB035897	AB082571
Bannoa ogasawarensis	JCM 10326 (TYPE)	AB035713	AB082570
Bannoa syzygii	JCM 10337 (TYPE)	AB035720	AB082573
Buckleyzyma armeniaca	CBS 8076 (TYPE)	AF444523	AF189920
Buckleyzyma aurantiaca	CBS 371 (TYPE)	AF444538	AF189921
Buckleyzyma kluyveri-nielii	CBS 7168 (TYPE)	AF444544	AF189988
Buckleyzyma phyllomatis	CBS 7198 (TYPE)	AF444515	AF189991
Buckleyzyma salicina	CBS 6983 (TYPE)	AF444511	AF189995
Cyphobasidium hypogymniicola	S-F264671	KU587700	KU587694
Cyphobasidium usneicola	S-F264675	KU587704	KU587699
Cyrenella elegans	CBS 274.82 (TYPE)	KJ778626	KJ708454
Cystobasidium benthicum	JCM 10901 (TYPE)	AB026001	AB026001
Cystobasidium calyptogenae	JCM 10899 (TYPE)	AB025996	AB025996
Cystobasidium fimentarium	DB1489	-	AY512843
Cystobasidium laryngis	JCM 10953 (TYPE)	AB078500	AB078500
Cystobasidium lysinophilum	JCM 5951 (TYPE)	AB078501	AB078501
Cystobasidium minutum	CBS 319 (TYPE)	AF190011	AF189945
Cystobasidium oligophagum	KM1106 (TYPE)	AB702968	AB702967
Cystobasidium pallidum	JCM 3780 (TYPE)	AB078492	AF189962
Cystobasidium pinicola	AS 2.2193 (TYPE)	AF444292	AF444293
Cystobasidium portillonenses	071209-Pi 2-frotapiedra-7-lev (TYPE)	JQ769323	JQ769312
Cystobasidium psychroaquaticum	CBS 11769 (TYPE)	KY103148	KY107444
Cystobasidium ritchiei	CBS 12324 (TYPE)	KY103149	KY107445
Cystobasidium slooffiae	JCM 10954 (TYPE)	AF444627	AF444722
Erythrobasidium elongatum	CBS8080 (TYPE)	AF444561	AF189983
Erythrobasidium hasegawianum	AS 2.1923 (TYPE)	AF444522	AF189899
Erythrobasidium yunnanensis	AS 2.209 (TYPE)	AB030353	AY335162
Hasegawazyma lactosa	CBS 5826 (TYPE)	NR 073295	NG 057668
Microsporomyces bloemfonteinensis	CBS 8598 (TYPE)	EU075189	EU075187
Microsporomyces hainanensis	CICC 33066 (TYPE)	KU296948	KU296947
Microsporomyces magnisporus	JCM 11898 (TYPE)	AB112078	AB111954
Microsporomyces orientis	CBS 8594 (TYPE)	HM559719	HM559718
Microsporomyces pini	CBS 107345 (TYPE)	EU075190	EU075188
Naohidea sebacea	CBS 8477 (TYPE)	DQ911616	DQ831020
Occultifur brasiliensis	UFMG-CM-Y376	KM248526	KM248525
Occultifur externus	JCM 10725 (TYPE)	AF444567	AF189910

Occultifur kilbournensis	NRRL Y-63695 (TYPE)	NR 155564	KP413160
Occultifur tropicalis	DMKU SE59 (TYPE)	NR 148062	-
Occultifur mephitis	EXF-6473	KX929057	-
Sakaguchia cladiensis	CBS 10878 (TYPE)	FJ008055	FJ008049
Sakaguchia dacryoidea	JCM 3795 (TYPE)	AF444597	AF189972
Sakaguchia lamellibrachii	CBS 9598 (TYPE)	AB025999	AB025999
Sakaguchia meli	CBS 10797 (TYPE)	FJ807683	KJ708452
Sakaguchia oryzae	AS2.2363 (TYPE)	AY335160	AY335161
Symmetrospora coprosmae	JCM 8772 (TYPE)	AF444577	AF189980
Symmetrospora foliicola	AS 2.2527 (TYPE)	AF444521	AF189984
Symmetrospora gracilis	JCM 2963 (TYPE)	AF444578	AF189985
Symmetrospora marina	JCM 3776 (TYPE)	AF444504	AF189944
Symmetrospora symmetrica	AS 2.2299 (TYPE)	AY364836	AY364836
Symmetrospora vermiculatus	JCM 10224 (TYPE)	AB030335	AF460176
Symmetrospora oryzicola	JCM 5299 (TYPE)	AF444546	AF189990
Microsporomyces pisutiana	SNI4A CKV1	MK491197	MK491270
Microsporomyces pisutiana	CSA5A CKV1	MK491194	MK491265
Microsporomyces pisutiana	Pol14e3 CKV	MK491198	MK491269
Microsporomyces pisutiana	LNV4A CKV1	MK491196	MK491266
Robertozyma ningxiaensis	CGMCC2.4451 (TYPE)	MK050392	-
Begerowomyces foliicola	CGMCC2.3164 (TYPE)	MK050394	-
Halobasidium xiangyangense	HBUAS51001 (TYPE)	MH209248	-
Queiroziella brasiliensis	BI02	MH244424	-
Cystobasidiopsis lophatheri	CBS 11272 (TYPE)	NR 144767	-
Cystobasidiopsis lactophilus	CBS 7725 (TYPE)	KY103120	KY107426
Cystobasidiopsis nirenbergiae	TUB F580 (TYPE)	NR 158377	-
Ballistosporomyces sasicola	CBS 7285 (TYPE)	NR 077095	NG 058696
Ballistosporomyces bomiensis	XZ33D1	JN620355	JN620355
Ballistosporomyces changbaiensis	CB266 (TYPE)	KP020105	KP020105
Kurtzmanomyces insolitus	CBS 8377	KY103926	KY108194
Septobasidium carestianum	DJM644	DQ241448	DQ241481
Septobasidium velutinum	DAH(024)	DQ241467	DQ242500
Septobasidium wilsonianum	DAH 037	DQ241469	DQ241502
Septobasidium taxodii	DAH 194c	DQ241466	DQ241499
Septobasidium pallidum	CLZhao 757	MG231816	
Septobasidium septobasidioides	DAH 032	DQ241461	DQ241494
Septobasidium gomezii	DAH 031	DQ241462	DQ241495
Septobasidium mariani	DAH 283b	DQ241456	DQ241489
Septobasidium pseudopedicellatum	DAH 044	DQ241460	DQ241493
Septobasidium sinuosum	DAH 036	DQ241464	DQ241497
Septobasidium alni	DAH FP3	DQ241441	DQ241474

Septobasidium apiculatum	DAH 064	DQ241442	DQ241475
Septobasidium arachnoideum	DAH 025	DQ241443	DQ241476
Septobasidium burtii	DAH 062	DQ241444	DQ241477
Septobasidium cavarae	DAH FP1	DQ241445	DQ241478
Septobasidium canescens	DAH 323	DQ241446	DQ241479
Septobasidium cokeri	DAH 061	DQ241449	DQ241482
Septobasidium ramorum	DAH 045a	DQ241450	DQ241483
Septobasidium fumigatum	DAH 005	DQ241451	DQ241484
Septobasidium grandisporum	DAH 065	DQ241453	DQ241486
Septobasidium griseum	DAH 008	DQ241455	DQ241488
Septobasidium michelianum	DAH FP5	DQ241457	DQ241490
Septobasidium pinicola	DAH 013	DQ241459	DQ241492
Septobasidium pilosum	DAH 020	DQ241458	DQ241491
Septobasidium meredithiae	DAH 257	DQ241465	DQ241498
Septobasidium westoni	DAH FP2001	DQ241468	DQ241501
Uredinella coccidiophaga	DAH 217c	DQ241471	DQ241504
Boekhoutia sterigmata	CGMCC2.4539	MK050371	MK050371
Pachnocybe ferruginea	DAH pf1	DQ241473	-

Table S2. The OTUs list with taxonomic identification and their read counts in each sample.

OTU ID	Phylum	Class	Order	Family	Genus	Species	Sample 9	Sample 10	Sample 11	Sample 12	Sample 13	Sample 14	Sample 15
OTU1	Basidiomycota	Cystobasidiomycetes	Cystobasidiales	Cystobasidiaceae	Cystobasidium	Cystobasidium sp	24307	17780	63813	43210	56886	149756	5879
OTU2	Basidiomycota	Pucciniomycetes	Septobasidiales	Septobasidiaceae	Septobasidium	Septobasidium carestianum	1842	73	5447	2297	2417	268608	7421
OTU3	Basidiomycota	Cystobasidiomycetes	Erythrobasidiales	Microsporomycetaceae	Microsporomyces	Microsporomyces sp	16928	2665	26441	3359	3182	9	5687
OTU4	Basidiomycota	Cystobasidiomycetes	Erythrobasidiales	Microsporomycetaceae	Microsporomyces	Microsporomyces ellipsoideus	4	0	2	55757	3	1	0
OTU5	Basidiomycota	Pucciniomycetes	Septobasidiales	Septobasidiaceae	Septobasidium	Septobasidium sp	1638	70	7223	5	8638	14901	15797
OTU6	Basidiomycota	Cystobasidiomycetes	Erythrobasidiales	Microsporomycetaceae	Microsporomyces	Cystobasidiomycetes sp	4	0	3	18323	4	3	2
OTU7	Basidiomycota	Cystobasidiomycetes	Erythrobasidiales	Microsporomycetaceae	Microsporomyces	Microsporomyces sp	28265	1	2	3	3	0	1
OTU8	Basidiomycota	Pucciniomycetes	Septobasidiales	Septobasidiaceae	Unidentified	Septobasidiaceae sp	334	1133	28963	987	529	5136	2427
OTU9	Basidiomycota	Cystobasidiomycetes	Cystobasidiales	Cystobasidiaceae	Occultifur	Occultifur mephitis	23920	2	369	10	175	28	4
OTU10	Basidiomycota	Pucciniomycetes	Septobasidiales	Septobasidiaceae	Unidentified	Septobasidiaceae sp	4	444	23716	1	5	19	16
OTU11	Basidiomycota	Cystobasidiomycetes	Erythrobasidiales	Microsporomycetaceae	Microsporomyces	Microsporomyces sp	9019	510	17938	1215	29917	5	1498
OTU12	Basidiomycota	Pucciniomycetes	Septobasidiales	Septobasidiaceae	Septobasidium	Septobasidium velutinum	2	97	7338	2	222	27	20839
OTU13	Basidiomycota	Pucciniomycetes	Septobasidiales	Septobasidiaceae	Septobasidium	Septobasidium pallidum	0	1	18651	0	0	16	8
OTU14	Basidiomycota	Cystobasidiomycetes	Erythrobasidiales	Buckleyzymaceae	Buckleyzyma	Buckleyzyma aurantiaca	1707	1827	2450	1302	2717	5564	427
OTU15	Basidiomycota	Cystobasidiomycetes	Erythrobasidiales	Unidentified	Unidentified	Erythrobasidiales sp	827	124	15696	3161	6777	1382	34
OTU16	Basidiomycota	Cystobasidiomycetes	Erythrobasidiales	Unidentified	Unidentified	Erythrobasidiales sp	396	12	7076	2472	2176	1134	12
OTU17	Basidiomycota	Pucciniomycetes	Septobasidiales	Septobasidiaceae	Unidentified	Septobasidiaceae sp	11	1	1163	5739	993	1249	27
OTU18	Basidiomycota	Cystobasidiomycetes	Erythrobasidiales	Microsporomycetaceae	Unidentified	Microsporomycetaceae sp	8646	1	22	402	18	2	0
OTU19	Basidiomycota	Cystobasidiomycetes	Erythrobasidiales	Erythrobasidiaceae	Erythrobasidium	Erythrobasidium sp	7633	37	542	68	1	1	1
OTU20	Basidiomycota	Cystobasidiomycetes	Erythrobasidiales	Erythrobasidiaceae	Erythrobasidium	Erythrobasidium sp	2495	4	103	12	0	0	0
OTU21	Basidiomycota	Cystobasidiomycetes	Erythrobasidiales	Unidentified	Unidentified	Erythrobasidiales sp	5317	0	8	886	807	5	90
OTU22	Basidiomycota	Tremellomycetes	Tremellales	Carcinomycetaceae	Carcinomyces	Carcinomyces polyporinus	1	2	2	0	3748	0	1
OTU23	Basidiomycota	Pucciniomycetes	Septobasidiales	Septobasidiaceae	Septobasidium	Septobasidium sp	635	0	3563	2	2	7	67
OTU24	Basidiomycota	Cystobasidiomycetes	Erythrobasidiales	Erythrobasidiaceae	Erythrobasidium	Erythrobasidium sp	9164	36	4	373	345	2	15
OTU25	Basidiomycota	Cystobasidiomycetes	Erythrobasidiales	Erythrobasidiaceae	Erythrobasidium	Erythrobasidium sp	6056	5	0	162	116	0	3

OTU26	Basidiomycota	Pucciniomycetes	Septobasidiales	Septobasidiaceae	Unidentified	Septobasidiaceae sp	0	0	3433	0	1	1	0
OTU27	Basidiomycota	Cystobasidiomycetes	Erythrobasidiales	Unidentified	Unidentified	Erythrobasidiales sp	5512	0	159	6	955	0	46
OTU28	Basidiomycota	Pucciniomycetes	Septobasidiales	Septobasidiaceae	Septobasidium	Septobasidium sp	29	0	7	2	6	4202	330
OTU29	Basidiomycota	Pucciniomycetes	Septobasidiales	Septobasidiaceae	Septobasidium	Septobasidium sp	0	0	6	2	3	3165	8
OTU30	Basidiomycota	Cystobasidiomycetes	Erythrobasidiales	Microsporomycetaceae	Microsporomyces	Lichenozyma pisutiana	7	0	17	9	17	7	3346
OTU31	Basidiomycota	Pucciniomycetes	Septobasidiales	Septobasidiaceae	Unidentified	Septobasidiaceae sp	15	0	778	1	3093	14	30
OTU32	Basidiomycota	Microbotryomycetes	Unidentified	Unidentified	Unidentified	Microbotryomycetes sp	1590	0	0	137	163	1	19
OTU33	Basidiomycota	Cystobasidiomycetes	Erythrobasidiales	Unidentified	Unidentified	Erythrobasidiales sp	2492	0	50	0	226	0	8
OTU34	Basidiomycota	Cystobasidiomycetes	Erythrobasidiales	Unidentified	Unidentified	Erythrobasidiales sp	102	230	10704	782	3	255	0
OTU35	Unidentified	Unidentified	Unidentified	Unidentified	Unidentified	Fungi sp	14	0	2324	0	0	0	0
OTU36	Basidiomycota	Pucciniomycetes	Septobasidiales	Septobasidiaceae	Septobasidium	Septobasidium sinuosum	0	0	5458	0	0	5	0
OTU37	Basidiomycota	Cystobasidiomycetes	Erythrobasidiales	Unidentified	Unidentified	Erythrobasidiales sp	14	31	2213	317	0	1	0
OTU38	Basidiomycota	Pucciniomycetes	Septobasidiales	Septobasidiaceae	Septobasidium	Septobasidium velutinum	1	0	125	1	3	41	2902
OTU39	Basidiomycota	Cystobasidiomycetes	Erythrobasidiales	Symmetrosporaceae	Symmetrospora	Symmetrospora sp	1896	0	617	0	3	0	0
OTU40	Basidiomycota	Cystobasidiomycetes	Erythrobasidiales	Erythrobasidiaceae	Erythrobasidium	Erythrobasidium sp	4409	3	13	8	634	236	142
OTU41	Basidiomycota	Pucciniomycetes	Platygloeales	Eocronartiaceae	Eocronartium	Eocronartium sp	1	0	15	4230	25	6	10
OTU42	Basidiomycota	Cystobasidiomycetes	Naohideales	Naohideaceae	Naohidea	Naohidea sp	2239	0	1	0	0	0	0
OTU43	Basidiomycota	Pucciniomycetes	Septobasidiales	Septobasidiaceae	Septobasidium	Septobasidium sp	22	0	373	0	120	4115	468
OTU44	Basidiomycota	Cystobasidiomycetes	Erythrobasidiales	Erythrobasidiaceae	Erythrobasidium	Erythrobasidium sp	1730	0	0	0	161	24	30
OTU45	Basidiomycota	Pucciniomycetes	Septobasidiales	Septobasidiaceae	Septobasidium	Septobasidium velutinum	0	0	2	0	0	10	2083
OTU46	Basidiomycota	Pucciniomycetes	Septobasidiales	Septobasidiaceae	Septobasidium	Septobasidium sp	1761	0	645	1	349	33	25
OTU47	Basidiomycota	Cystobasidiomycetes	Erythrobasidiales	Unidentified	Unidentified	Erythrobasidiales sp	1264	1	6	111	572	1	50
OTU48	Basidiomycota	Tremellomycetes	Tremellales	Cuniculitremaceae	Kockovaella	Kockovaella sp	122	26	1370	0	0	0	0
OTU49	Basidiomycota	Pucciniomycetes	Septobasidiales	Septobasidiaceae	Septobasidium	Septobasidium velutinum	0	0	14	0	1257	8	6
OTU50	Basidiomycota	Microbotryomycetes	Sporidiobolales	Sporidiobolaceae	Sporobolomyces	Sporobolomyces sp	1895	0	1	0	1	1	5
OTU51	Basidiomycota	Pucciniomycetes	Septobasidiales	Septobasidiaceae	Septobasidium	Septobasidium sp	1358	1	31	3	2	9	7
OTU52	Basidiomycota	Cystobasidiomycetes	Erythrobasidiales	Erythrobasidiaceae	Erythrobasidium	Erythrobasidium sp	8	0	1371	723	0	0	0
OTU53	Basidiomycota	Tremellomycetes	Tremellales	Bulleribasidiaceae	Hannaella	Hannaella kunmingensis	0	3	329	30	340	181	0
OTU54	Basidiomycota	Pucciniomycetes	Septobasidiales	Septobasidiaceae	Septobasidium	Septobasidium sp	1	0	1025	0	1	0	0
OTU55	Basidiomycota	Pucciniomycetes	Septobasidiales	Septobasidiaceae	Unidentified	Septobasidiaceae sp	2	0	22	420	1081	16	14
OTU56	Basidiomycota	Pucciniomycetes	Septobasidiales	Septobasidiaceae	Unidentified	Septobasidiaceae sp	0	0	1841	2	1	1	0

OTU57	Basidiomycota	Pucciniomycetes	Septobasidiales	Septobasidiaceae	Unidentified	Septobasidiaceae sp	959	0	19	4	20	12	29
OTU58	Basidiomycota	Cystobasidiomycetes	Erythrobasidiales	Microsporomycetaceae	Unidentified	Microsporomycetaceae sp	489	46	1814	5	1344	0	0
OTU59	Basidiomycota	Pucciniomycetes	Septobasidiales	Septobasidiaceae	Septobasidium	Septobasidium velutinum	1	0	999	0	0	0	0
OTU60	Basidiomycota	Pucciniomycetes	Septobasidiales	Septobasidiaceae	Septobasidium	Septobasidium velutinum	0	0	677	0	0	437	1
OTU61	Basidiomycota	Cystobasidiomycetes	Cystobasidiales	Cystobasidiaceae	Cystobasidium	Cystobasidium sp	11	3	38	29	33	883	6
OTU62	Basidiomycota	Pucciniomycetes	Septobasidiales	Septobasidiaceae	Septobasidium	Septobasidium carestianum	4	2	8	5	1	1135	16
OTU63	Basidiomycota	Microbotryomycetes	Sporidiobolales	Sporidiobolaceae	Sporobolomyces	Sporobolomyces sp	741	0	0	0	0	0	13
OTU64	Basidiomycota	Cystobasidiomycetes	Erythrobasidiales	Microsporomycetaceae	Unidentified	Microsporomycetaceae sp	795	1	3	0	3	0	64
OTU65	Basidiomycota	Cystobasidiomycetes	Erythrobasidiales	Erythrobasidiaceae	Erythrobasidium	Erythrobasidium sp	434	0	342	308	1	0	0
OTU66	Basidiomycota	Cystobasidiomycetes	Erythrobasidiales	Buckleyzymaceae	Buckleyzyma	Buckleyzyma aurantiaca	1	0	6	1	3	783	0
OTU67	Basidiomycota	Pucciniomycetes	Septobasidiales	Septobasidiaceae	Septobasidium	Septobasidium velutinum	135	0	847	3	4	2	1
OTU68	Basidiomycota	Pucciniomycetes	Platygloeales	Platygloeaceae	Platygloea	Platygloea disciformis	1	0	8	19	1347	7	10
OTU69	Basidiomycota	Microbotryomycetes	Sporidiobolales	Sporidiobolaceae	Sporobolomyces	Sporobolomyces sp	776	0	0	0	0	1	0
OTU70	Basidiomycota	Cystobasidiomycetes	Erythrobasidiales	Erythrobasidiaceae	Erythrobasidium	Erythrobasidium sp	1120	0	128	48	2	0	0
OTU71	Unidentified	Unidentified	Unidentified	Unidentified	Unidentified	Fungi sp	0	0	3	0	296	204	0
OTU72	Basidiomycota	Pucciniomycetes	Septobasidiales	Septobasidiaceae	Septobasidium	Septobasidium mariani	1	0	4	0	546	2	3
OTU73	Basidiomycota	Cystobasidiomycetes	Erythrobasidiales	Microsporomycetaceae	Microsporomyces	Microsporomyces pini	1	199	0	1884	2	2	0
OTU74	Basidiomycota	Pucciniomycetes	Septobasidiales	Septobasidiaceae	Septobasidium	Septobasidium pseudopedicellatum	1	0	14	133	532	9	5
OTU75	Basidiomycota	Microbotryomycetes	Sporidiobolales	Unidentified	Unidentified	Sporidiobolales sp	0	0	0	0	557	1	0
OTU76	Basidiomycota	Cystobasidiomycetes	Erythrobasidiales	Microsporomycetaceae	Microsporomyces	Microsporomyces sp	1	0	0	674	0	1	0
OTU77	Basidiomycota	Tremellomycetes	Tremellales	Trimorphomycetaceae	Saitozyma	Saitozyma sp	262	20	161	1	0	1	0
OTU78	Basidiomycota	Cystobasidiomycetes	Erythrobasidiales	Erythrobasidiaceae	Erythrobasidium	Erythrobasidium sp	0	0	476	184	0	0	0
OTU79	Basidiomycota	Pucciniomycetes	Septobasidiales	Septobasidiaceae	Septobasidium	Septobasidium velutinum	0	0	0	0	0	0	493
OTU80	Ascomycota	Arthoniomycetes	Arthoniales	Roccellaceae	Gyrographa	Gyrographa gyrocarpa	6	1	496	2	0	0	0
OTU81	Basidiomycota	Cystobasidiomycetes	Cystobasidiales	Cystobasidiaceae	Cystobasidium	Cystobasidium sp	1	505	2	0	0	2	0
OTU82	Basidiomycota	Pucciniomycetes	Septobasidiales	Septobasidiaceae	Septobasidium	Septobasidium carestianum	0	0	2	1	418	5	69
OTU83	Basidiomycota	Microbotryomycetes	Microbotryomycetes	Microbotryomycetes	Colacogloea	Colacogloea sp	1150	0	2	0	0	1	0
OTU84	Basidiomycota	Cystobasidiomycetes	Erythrobasidiales	Erythrobasidiaceae	Erythrobasidium	Erythrobasidium sp	708	0	3	4	2	171	0
OTU85	Basidiomycota	Microbotryomycetes	Microbotryomycetes	Microbotryomycetes	Colacogloea	Colacogloea sp	477	0	0	0	0	0	0

OTU86	Basidiomycota	Cystobasidiomycetes	Erythrobasidiales	Erythrobasidiaceae	Erythrobasidium	Erythrobasidium sp	103	0	62	75	1	0	0
OTU87	Basidiomycota	Cystobasidiomycetes	Erythrobasidiales	Unidentified	Unidentified	Cystobasidiales sp	486	0	334	1	0	0	0
OTU88	Basidiomycota	Pucciniomycetes	Septobasidiales	Septobasidiaceae	Septobasidium	Septobasidium sp	0	0	1	0	0	439	0
OTU89	Basidiomycota	Cystobasidiomycetes	Erythrobasidiales	Erythrobasidiaceae	Erythrobasidium	Erythrobasidium sp	142	0	2	709	5	0	0
OTU90	Basidiomycota	Cystobasidiomycetes	Erythrobasidiales	Symmetrosporaceae	Symmetrospora	Symmetrospora sp	0	0	364	0	197	0	0
OTU91	Basidiomycota	Pucciniomycetes	Septobasidiales	Septobasidiaceae	Septobasidium	Septobasidium pseudopedicellatum	338	0	11	2	1	6	5
OTU92	Basidiomycota	Pucciniomycetes	Septobasidiales	Septobasidiaceae	Septobasidium	Septobasidium pallidum	0	0	8	343	2	6	6
OTU93	Basidiomycota	Pucciniomycetes	Septobasidiales	Septobasidiaceae	Septobasidium	Septobasidium sp	0	0	346	0	4	1	2
OTU94	Basidiomycota	Cystobasidiomycetes	Erythrobasidiales	Microsporomycetaceae	Microsporomyces	Microsporomyces sp	0	0	2	84	631	0	1
OTU95	Unidentified	Unidentified	Unidentified	Unidentified	Unidentified	Fungi sp	0	139	206	1	2	5	0
OTU96	Basidiomycota	Cystobasidiomycetes	Unidentified	Unidentified	Unidentified	Cystobasidiomycetes sp	449	0	0	1	1	1	0
OTU97	Basidiomycota	Pucciniomycetes	Septobasidiales	Septobasidiaceae	Septobasidium	Septobasidium pseudopedicellatum	0	0	303	5	1	0	0
OTU98	Basidiomycota	Cystobasidiomycetes	Erythrobasidiales	Erythrobasidiaceae	Erythrobasidium	Erythrobasidium sp	375	0	24	2	1	0	0
OTU99	Basidiomycota	Pucciniomycetes	Septobasidiales	Septobasidiaceae	Septobasidium	Septobasidium sp	0	0	276	0	0	0	0
OTU100	Basidiomycota	Tremellomycetes	Tremellales	Tremellaceae	Cryptococcus	Cryptococcus sp	90	142	1	0	1	0	0
OTU101	Basidiomycota	Cystobasidiomycetes	Erythrobasidiales	Unidentified	Unidentified	Erythrobasidiales sp	1	0	350	0	0	0	0
OTU102	Basidiomycota	Cystobasidiomycetes	Erythrobasidiales	Erythrobasidiaceae	Bannoa	Bannoa ogasawarensis	793	0	2	47	159	0	42
OTU103	Basidiomycota	Tremellomycetes	Tremellales	Syzygosporaceae	Syzygospora	Syzygospora effibulata	61	60	187	0	0	0	0
OTU104	Basidiomycota	Pucciniomycetes	Septobasidiales	Septobasidiaceae	Unidentified	Septobasidiaceae sp	0	0	280	0	0	0	0
OTU105	Basidiomycota	Pucciniomycetes	Septobasidiales	Septobasidiaceae	Septobasidium	Septobasidium sp	0	0	2	0	265	3	1
OTU106	Basidiomycota	Cystobasidiomycetes	Erythrobasidiales	Microsporomycetaceae	Unidentified	Microsporomycetaceae sp	0	0	0	290	0	1	0
OTU107	Basidiomycota	Pucciniomycetes	Septobasidiales	Septobasidiaceae	Unidentified	Septobasidiaceae sp	274	0	5	5	12	11	4
OTU108	Basidiomycota	Tremellomycetes	Tremellales	Cuniculitremaceae	Kockovaella	Kockovaella sp	0	0	261	1	0	0	0
OTU109	Basidiomycota	Pucciniomycetes	Septobasidiales	Septobasidiaceae	Septobasidium	Septobasidium velutinum	0	0	1	0	0	1	253
OTU110	Basidiomycota	Pucciniomycetes	Septobasidiales	Septobasidiaceae	Septobasidium	Septobasidium velutinum	0	0	286	0	1	0	0
OTU111	Basidiomycota	Cystobasidiomycetes	Erythrobasidiales	Symmetrosporaceae	Symmetrospora	Symmetrospora symmetrica	14	0	3	2	355	2	37
OTU112	Basidiomycota	Cystobasidiomycetes	Erythrobasidiales	Erythrobasidiaceae	Erythrobasidium	Erythrobasidium sp	217	0	0	0	0	29	0
OTU113	Basidiomycota	Cystobasidiomycetes	Erythrobasidiales	Erythrobasidiaceae	Bannoa	Bannoa ogasawarensis	236	0	0	378	127	1	0
OTU114	Basidiomycota	Cystobasidiomycetes	Cystobasidiales	Cystobasidiaceae	Occultifur	Occultifur mephitis	4	0	1	0	246	1	0
OTU115	Basidiomycota	Cystobasidiomycetes	Erythrobasidiales	Erythrobasidiaceae	Bannoa	Bannoa ogasawarensis	330	62	1	113	118	1	10

OTU116	Basidiomycota	Cystobasidiomycetes	Erythrobasidiales	Erythrobasidiaceae	Erythrobasidium	Erythrobasidium sp	1	1	2	499	0	0	0
OTU117	Basidiomycota	Agaricomycetes	Agaricales	Marasmiaceae	Marasmius	Marasmius sp	0	0	0	686	0	0	1
OTU118	Basidiomycota	Pucciniomycetes	Septobasidiales	Septobasidiaceae	Septobasidium	Septobasidium velutinum	0	0	196	0	0	0	0
OTU119	Basidiomycota	Cystobasidiomycetes	Erythrobasidiales	Erythrobasidiaceae	Erythrobasidium	Erythrobasidium sp	44	0	0	271	0	0	0
OTU120	Basidiomycota	Cystobasidiomycetes	Erythrobasidiales	Unidentified	Unidentified	Erythrobasidiales sp	89	0	6767	135	1	74	0
OTU121	Basidiomycota	Pucciniomycetes	Septobasidiales	Septobasidiaceae	Septobasidium	Septobasidium velutinum	0	0	0	0	0	1	216
OTU122	Basidiomycota	Tremellomycetes	Tremellales	Carcinomycetaceae	Carcinomyces	Carcinomyces polyporinus	0	128	2	0	55	1	0
OTU123	Basidiomycota	Cystobasidiomycetes	Cystobasidiales	Cystobasidiaceae	Occultifur	Occultifur mephitis	218	0	4	0	4	0	0
OTU124	Ascomycota	Arthoniomycetes	Arthoniales	Opegraphaceae	Opegrapha	Opegrapha vermicellifera	130	0	44	0	1	0	0
OTU125	Basidiomycota	Cystobasidiomycetes	Erythrobasidiales	Erythrobasidiaceae	Bannoa	Bannoa ogasawarensis	348	0	0	1	0	0	0
OTU126	Basidiomycota	Cystobasidiomycetes	Cystobasidiales	Cystobasidiaceae	Occultifur	Occultifur sp	112	0	0	0	0	0	0
OTU127	Basidiomycota	Cystobasidiomycetes	Erythrobasidiales	Unidentified	Unidentified	Erythrobasidiales sp	86	0	1716	11	2	346	14
OTU128	Basidiomycota	Cystobasidiomycetes	Erythrobasidiales	Unidentified	Unidentified	Erythrobasidiales sp	1	0	3	111	943	1	11
OTU129	Basidiomycota	Cystobasidiomycetes	Erythrobasidiales	Erythrobasidiaceae	Bannoa	Bannoa ogasawarensis	64	13	0	56	46	0	1
OTU130	Basidiomycota	Microbotryomycetes	Sporidiobolales	Sporidiobolaceae	Rhodosporidiobolus	Rhodosporidiobolus sp	0	0	0	214	0	0	0
OTU131	Basidiomycota	Pucciniomycetes	Septobasidiales	Septobasidiaceae	Septobasidium	Septobasidium pseudopedicellatum	0	0	1	0	122	1	1
OTU132	Basidiomycota	Cystobasidiomycetes	Erythrobasidiales	Microsporomycetaceae	Microsporomyces	Microsporomyces sp	0	0	0	25	153	0	0
OTU133	Basidiomycota	Tremellomycetes	Tremellales	Unidentified	Unidentified	Tremellales sp	99	0	0	0	0	0	0
OTU134	Basidiomycota	Tremellomycetes	Tremellales	Cuniculitremaceae	Kockovaella	Kockovaella prillingeri	2	0	260	1	0	0	1
OTU135	Basidiomycota	Cystobasidiomycetes	Erythrobasidiales	Erythrobasidiaceae	Erythrobasidium	Erythrobasidium sp	0	0	0	83	0	0	0
OTU136	Basidiomycota	Agaricomycetes	Agaricales	Inocybaceae	Inocybe	Inocybe sp	0	0	134	0	0	0	0
OTU137	Basidiomycota	Cystobasidiomycetes	Erythrobasidiales	Erythrobasidiaceae	Erythrobasidium	Erythrobasidium sp	0	0	0	145	0	0	0
OTU138	Basidiomycota	Pucciniomycetes	Septobasidiales	Septobasidiaceae	Septobasidium	Septobasidium pallidum	0	0	0	0	0	98	5
OTU139	Basidiomycota	Cystobasidiomycetes	Naohideales	Naohideaceae	Naohidea	Naohidea sebacea	92	0	0	0	0	0	0
OTU140	Basidiomycota	Cystobasidiomycetes	Erythrobasidiales	Sakaguchiaceae	Sakaguchia	Sakaguchia lamellibrachiae	0	0	0	106	0	0	0
OTU141	Basidiomycota	Tremellomycetes	Tremellales	Unidentified	Unidentified	Tremellales sp	10	0	79	0	0	0	0
OTU142	Basidiomycota	Cystobasidiomycetes	Naohideales	Naohideaceae	Naohidea	Naohidea sebacea	0	0	0	83	0	0	0
OTU143	Basidiomycota	Cystobasidiomycetes	Erythrobasidiales	Sakaguchiaceae	Sakaguchia	Sakaguchia dacryoidea	125	0	0	0	0	0	0
OTU144	Basidiomycota	Microbotryomycetes	Sporidiobolales	Sporidiobolaceae	Rhodosporidiobolus	Rhodosporidiobolus sp	0	0	0	47	0	0	0
OTU145	Unidentified	Unidentified	Unidentified	Unidentified	Unidentified	Fungi sp	0	0	0	0	58	2	0

OTU146	Basidiomycota	Pucciniomycetes	Septobasidiales	Septobasidiaceae	Septobasidium	Septobasidium pallidum	0	0	1	0	0	76	18
OTU147	Basidiomycota	Cystobasidiomycetes	Erythrobasidiales	Erythrobasidiaceae	Erythrobasidium	Erythrobasidium hasegawianum	0	0	94	0	0	0	0
OTU148	Basidiomycota	Pucciniomycetes	Septobasidiales	Septobasidiaceae	Septobasidium	Septobasidium ramorum	0	0	0	0	2	0	57
OTU149	Basidiomycota	Microbotryomycetes	Sporidiobolales	Sporidiobolaceae	Rhodosporidiobolus	Rhodosporidiobolus fluvialis	10	0	0	96	0	0	0
OTU150	Basidiomycota	Tremellomycetes	Tremellales	Bulleribasidiaceae	Hannaella	Hannaella phetchabunensis	0	0	49	0	0	0	0
OTU151	Basidiomycota	Pucciniomycetes	Platygloeales	Eocronartiaceae	Eocronartium	Eocronartium sp	0	0	0	62	0	0	0
OTU152	Basidiomycota	Cystobasidiomycetes	Erythrobasidiales	Erythrobasidiaceae	Bannoa	Bannoa sp	1	0	3	0	0	68	0
OTU153	Basidiomycota	Cystobasidiomycetes	Erythrobasidiales	Sakaguchiaceae	Sakaguchia	Sakaguchia dacryoidea	31	0	0	0	0	0	0
OTU154	Basidiomycota	Cystobasidiomycetes	Cystobasidiales	Cystobasidiaceae	Occultifur	Occultifur sp	73	0	0	0	0	0	0
OTU155	Unidentified	Unidentified	Unidentified	Unidentified	Unidentified	Fungi sp	0	0	0	0	54	0	1
OTU156	Basidiomycota	Cystobasidiomycetes	Erythrobasidiales	Unidentified	Unidentified	Erythrobasidiales sp	0	0	0	0	64	0	0
OTU157	Basidiomycota	Tremellomycetes	Tremellales	Cuniculitremaceae	Kockovaella	Kockovaella sp	6	0	14	19	1	0	0
OTU158	Basidiomycota	Cystobasidiomycetes	Cystobasidiales	Cystobasidiaceae	Cystobasidium	Cystobasidium sp	58	0	1	0	2	5	0
OTU159	Basidiomycota	Pucciniomycetes	Septobasidiales	Septobasidiaceae	Septobasidium	Septobasidium pallidum	0	0	88	0	0	1	0
OTU160	Basidiomycota	Pucciniomycetes	Septobasidiales	Septobasidiaceae	Unidentified	Septobasidiaceae sp	0	0	1	100	0	0	1
OTU161	Basidiomycota	Pucciniomycetes	Septobasidiales	Septobasidiaceae	Septobasidium	Septobasidium carestianum	0	0	0	0	46	0	1
OTU162	Basidiomycota	Microbotryomycetes	Microbotryomycetes	Chrysozymaceae	Oberwinklerozyma	Oberwinklerozyma sp	33	24	7	0	0	0	0
OTU163	Basidiomycota	Pucciniomycetes	Septobasidiales	Septobasidiaceae	Septobasidium	Septobasidium kameii	0	0	0	0	0	0	44
OTU164	Basidiomycota	Cystobasidiomycetes	Erythrobasidiales	Erythrobasidiaceae	Erythrobasidium	Erythrobasidium hasegawianum	0	0	0	20	0	0	0
OTU165	Basidiomycota	Microbotryomycetes	Microbotryomycetes	Chrysozymaceae	Bannozyma	Bannozyma sp	0	0	0	66	0	1	0
OTU166	Basidiomycota	Tremellomycetes	Tremellales	Unidentified	Unidentified	Tremellales sp	0	36	0	0	0	0	0
OTU167	Basidiomycota	Cystobasidiomycetes	Erythrobasidiales	Symmetrosporaceae	Symmetrospora	Symmetrospora sp	36	0	11	0	0	0	0
OTU168	Basidiomycota	Pucciniomycetes	Platygloeales	Eocronartiaceae	Eocronartium	Eocronartium sp	0	0	0	0	0	0	39
OTU169	Basidiomycota	Pucciniomycetes	Septobasidiales	Septobasidiaceae	Septobasidium	Septobasidium velutinum	0	0	0	0	0	0	31
OTU170	Basidiomycota	Cystobasidiomycetes	Erythrobasidiales	Sakaguchiaceae	Sakaguchia	Sakaguchia lamellibrachiae	0	0	0	33	0	0	0
OTU171	Basidiomycota	Pucciniomycetes	Septobasidiales	Septobasidiaceae	Septobasidium	Septobasidium carestianum	0	0	0	0	0	0	37
OTU172	Basidiomycota	Tremellomycetes	Tremellales	Trimorphomycetaceae	Saitozyma	Saitozyma sp	1	0	25	0	0	0	0
OTU173	Basidiomycota	Cystobasidiomycetes	Erythrobasidiales	Unidentified	Unidentified	Erythrobasidiales sp	991	0	0	2	1	2	0

OTU174	Basidiomycota	Pucciniomycetes	Septobasidiales	Septobasidiaceae	Septobasidium	Septobasidium velutinum	0	0	0	0	0	0	33
OTU175	Basidiomycota	Tremellomycetes	Tremellales	Unidentified	Unidentified	Tremellales sp	1	26	0	0	0	0	0
OTU176	Basidiomycota	Pucciniomycetes	Septobasidiales	Septobasidiaceae	Septobasidium	Septobasidium sp	55	0	1	1	0	3	10
OTU177	Basidiomycota	Cystobasidiomycetes	Unidentified	Unidentified	Unidentified	Cystobasidiomycetes sp	0	0	2	0	0	1	27
OTU178	Basidiomycota	Pucciniomycetes	Septobasidiales	Septobasidiaceae	Unidentified	Septobasidiaceae sp	0	0	1	0	0	0	31
OTU179	Basidiomycota	Cystobasidiomycetes	Erythrobasidiales	Erythrobasidiaceae	Erythrobasidium	Erythrobasidium sp	0	0	0	36	0	0	0
OTU180	Basidiomycota	Cystobasidiomycetes	Erythrobasidiales	Erythrobasidiaceae	Erythrobasidium	Erythrobasidium sp	1	0	65	0	0	0	0
OTU181	Basidiomycota	Tremellomycetes	Tremellales	Bulleribasidiaceae	Hannaella	Hannaella sp	0	0	0	31	0	0	0
OTU182	Basidiomycota	Agaricomycetes	Geastrales	Geastraceae	Geastrum	Geastrum triplex	0	0	0	8	29	0	0
OTU183	Basidiomycota	Tremellomycetes	Tremellales	Unidentified	Unidentified	Tremellales sp	1	18	1	0	0	0	0
OTU184	Basidiomycota	Pucciniomycetes	Septobasidiales	Septobasidiaceae	Septobasidium	Septobasidium velutinum	0	0	0	0	7	0	0
OTU185	Basidiomycota	Microbotryomycetes	Sporidiobolales	Sporidiobolaceae	Rhodosporidiobolus	Rhodosporidiobolus fluvialis	5	0	0	26	0	0	0
OTU186	Basidiomycota	Cystobasidiomycetes	Erythrobasidiales	Unidentified	Unidentified	Erythrobasidiales sp	0	0	124	4	0	0	0
OTU187	Basidiomycota	Tremellomycetes	Tremellales	Bulleribasidiaceae	Hannaella	Hannaella sp	0	0	0	0	17	0	0
OTU188	Basidiomycota	Tremellomycetes	Tremellales	Tremellaceae	Cryptococcus	Cryptococcus sp	2	17	0	0	0	0	0
OTU189	Basidiomycota	Cystobasidiomycetes	Erythrobasidiales	Erythrobasidiaceae	Erythrobasidium	Erythrobasidium hasegawianum	0	0	9	0	0	0	0
OTU190	Basidiomycota	Microbotryomycetes	Microbotryomycetes	Chrysozymaceae	Bannozyma	Bannozyma sp	0	0	0	12	0	2	0
OTU191	Basidiomycota	Pucciniomycetes	Septobasidiales	Septobasidiaceae	Septobasidium	Septobasidium pallidum	0	1	138	0	0	0	0
OTU192	Basidiomycota	Pucciniomycetes	Septobasidiales	Septobasidiaceae	Septobasidium	Septobasidium sp	2	0	0	0	1	0	0
OTU193	Basidiomycota	Cystobasidiomycetes	Erythrobasidiales	Erythrobasidiaceae	Bannoa	Bannoa sp	0	0	0	0	0	15	0
OTU195	Basidiomycota	Pucciniomycetes	Septobasidiales	Septobasidiaceae	Septobasidium	Septobasidium pseudopedicellatum	1	0	0	0	0	0	24
OTU196	Basidiomycota	Cystobasidiomycetes	Erythrobasidiales	Symmetrosporaceae	Symmetrospora	Symmetrospora symmetrica	0	0	0	0	16	0	0
OTU197	Basidiomycota	Microbotryomycetes	Sporidiobolales	Sporidiobolaceae	Sporobolomyces	Sporobolomyces sp	12	0	11	0	18	0	0
OTU198	Basidiomycota	Tremellomycetes	Tremellales	Unidentified	Unidentified	Tremellales sp	7	3	13	23	24	2	0
OTU199	Basidiomycota	Cystobasidiomycetes	Erythrobasidiales	Unidentified	Unidentified	Erythrobasidiales sp	0	0	19	0	0	0	0
OTU200	Basidiomycota	Tremellomycetes	Tremellales	Unidentified	Unidentified	Tremellales sp	0	0	13	0	0	0	0
OTU201	Basidiomycota	Tremellomycetes	Tremellales	Cuniculitremaceae	Kockovaella	Kockovaella ogasawarensis	0	0	0	18	0	0	0
OTU202	Basidiomycota	Microbotryomycetes	Sporidiobolales	Unidentified	Unidentified	Sporidiobolales sp	0	22	0	0	0	0	0
OTU203	Basidiomycota	Microbotryomycetes	Sporidiobolales	Sporidiobolaceae	Sporobolomyces	Sporobolomyces sp	1	0	0	0	0	0	0

OTU204	Unidentified	Unidentified	Unidentified	Unidentified	Unidentified	Fungi sp	0	0	1048	6	0	0	0
OTU205	Basidiomycota	Pucciniomycetes	Septobasidiales	Septobasidiaceae	Septobasidium	Septobasidium velutinum	0	0	15	0	0	0	0
OTU206	Basidiomycota	Cystobasidiomycetes	Cystobasidiales	Cystobasidiaceae	Cystobasidium	Cystobasidium sp	4	8	21	0	0	1	1
OTU207	Basidiomycota	Tremellomycetes	Tremellales	Carcinomycetaceae	Carcinomyces	Carcinomyces sp	0	14	0	0	0	0	0
OTU209	Basidiomycota	Microbotryomycetes	Sporidiobolales	Sporidiobolaceae	Sporobolomyces	Sporobolomyces sp	27	0	0	0	0	0	0
OTU210	Basidiomycota	Cystobasidiomycetes	Cystobasidiales	Cystobasidiaceae	Cystobasidium	Cystobasidium sp	5	0	151	7	0	0	0
OTU211	Basidiomycota	Tremellomycetes	Tremellales	Tremellaceae	Tremella	Tremella diploschistina	9	0	6	2	4	0	0
OTU212	Basidiomycota	Cystobasidiomycetes	Cystobasidiales	Cystobasidiaceae	Cystobasidium	Cystobasidium sp	0	0	0	1	23	0	0
OTU213	Basidiomycota	Tremellomycetes	Tremellales	Cuniculitremaceae	Kockovaella	Kockovaella sp	0	2	3	0	1	0	0
OTU214	Basidiomycota	Microbotryomycetes	Sporidiobolales	Sporidiobolaceae	Rhodotorula	Rhodotorula sp	7	0	0	9	0	0	0
OTU215	Basidiomycota	Pucciniomycetes	Septobasidiales	Septobasidiaceae	Septobasidium	Septobasidium mariani	0	0	0	0	7	0	0
OTU216	Basidiomycota	Pucciniomycetes	Septobasidiales	Septobasidiaceae	Unidentified	Septobasidiaceae sp	0	0	80	1	0	1	0
OTU217	Ascomycota	Lecanoromycetes	Lecanorales	Cladoniaceae	Cladonia	Cladonia sp	0	2	73	1	6	0	0
OTU218	Basidiomycota	Cystobasidiomycetes	Cystobasidiales	Cystobasidiaceae	Cystobasidium	Cystobasidium sp	4	0	19	0	0	0	0
OTU219	Basidiomycota	Tremellomycetes	Tremellales	Cuniculitremaceae	Kockovaella	Kockovaella sp	10	1	1	0		0	5
OTU220	Basidiomycota	Microbotryomycetes	Sporidiobolales	Sporidiobolaceae	Rhodotorula	Rhodotorula paludigena	16	0	0	0	0	0	0
OTU221	Basidiomycota	Microbotryomycetes	Microbotryomycetes	Microbotryomycetes	Curvibasidium	Curvibasidium pallidicorallinum	25	0	1	0	0	0	0
OTU222	Basidiomycota	Cystobasidiomycetes	Naohideales	Unidentified	Unidentified	Naohideales sp	0	0	0	25	0	0	1
OTU223	Basidiomycota	Microbotryomycetes	Sporidiobolales	Unidentified	Unidentified	Sporidiobolales sp	0	0	0	0	10	0	0
OTU224	Basidiomycota	Pucciniomycetes	Septobasidiales	Septobasidiaceae	Septobasidium	Septobasidium velutinum	0	0	8	0	0	0	0
OTU225	Basidiomycota	Microbotryomycetes	Sporidiobolales	Sporidiobolaceae	Sporobolomyces	Sporobolomyces sp	2	0	4	0	3	0	0
OTU226	Basidiomycota	Tremellomycetes	Tremellales	Trimorphomycetaceae	Carlosrosaea	Carlosrosaea sp	0	0	5	0	9	0	2
OTU227	Basidiomycota	Tremellomycetes	Tremellales	Cuniculitremaceae	Kockovaella	Kockovaella mexicana	0	0	10	0	0	0	0
OTU228	Basidiomycota	Microbotryomycetes	Sporidiobolales	Sporidiobolaceae	Rhodotorula	Rhodotorula paludigena	4	0	0	0	0	0	0
OTU229	Basidiomycota	Pucciniomycetes	Septobasidiales	Septobasidiaceae	Septobasidium	Septobasidium carestianum	0	0	0	0	0	36	0
OTU230	Basidiomycota	Tremellomycetes	Tremellales	Cuniculitremaceae	Kockovaella	Kockovaella calophylli	1	0	3	0	0	0	0
OTU231	Basidiomycota	Pucciniomycetes	Septobasidiales	Septobasidiaceae	Unidentified	Unidentified	0	0	0	0	1	0	3
OTU232	Basidiomycota	Agaricomycetes	Hymenochaetales	Schizoporaceae	Xylodon	Xylodon crustosus	6	1	0	0	0	0	0
OTU233	Basidiomycota	Pucciniomycetes	Septobasidiales	Septobasidiaceae	Septobasidium	Septobasidium sp	0	0	0	0	0	36	37
OTU234	Basidiomycota	Microbotryomycetes	Microbotryomycetes	Chrysozymaceae	Oberwinklerozyma	Oberwinklerozyma silvestris	1	0	12	0	0	0	0

OTU235	Basidiomycota	Cystobasidiomycetes	Erythrobasidiales	Erythrobasidiaceae	Erythrobasidium	Erythrobasidium sp	0	0	9	0	0	0	0
OTU236	Basidiomycota	Cystobasidiomycetes	Erythrobasidiales	Erythrobasidiaceae	Erythrobasidium	Erythrobasidium sp	0	0	0	3	0	0	0
OTU237	Basidiomycota	Tremellomycetes	Tremellales	Cuniculitremaceae	Kockovaella	Kockovaella sp	0	0	6	0	0	0	0
OTU238	Basidiomycota	Tremellomycetes	Tremellales	Cuniculitremaceae	Kockovaella	Kockovaella prillingeri	0	0	7	3	0	0	0
OTU239	Basidiomycota	Pucciniomycetes	Septobasidiales	Septobasidiaceae	Unidentified	Septobasidiaceae sp	0	0	6	0	0	0	0
OTU240	Basidiomycota	Tremellomycetes	Tremellales	Cuniculitremaceae	Kockovaella	Kockovaella sp	0	0	2	2	0	0	0
OTU241	Basidiomycota	Pucciniomycetes	Septobasidiales	Septobasidiaceae	Unidentified	Septobasidiaceae sp	0	0	8	0	0	0	0
OTU242	Basidiomycota	Pucciniomycetes	Septobasidiales	Septobasidiaceae	Septobasidium	Septobasidium carestianum	1	0	11	0	0	0	0
OTU243	Basidiomycota	Microbotryomycetes	Microbotryomycetes	Chrysozymaceae	Spencerozyma	Spencerozyma sp	22	0	0	0	0	0	0
OTU244	Basidiomycota	Tremellomycetes	Tremellales	Rhynchogastremataceae	Papiliotrema	Papiliotrema aurea	8	0	1	0	1	0	0
OTU245	Basidiomycota	Cystobasidiomycetes	Erythrobasidiales	Unidentified	Unidentified	Erythrobasidiales sp	0	0	0	0	5	0	0
OTU246	Basidiomycota	Microbotryomycetes	Microbotryomycetes	Heterogastridiaceae	Colacogloea	Colacogloea cycloclastica	0	0	5	2	0	0	0
OTU247	Basidiomycota	Agaricomycetes	Agaricales	Inocybaceae	Inocybe	Inocybe sp	0	0	1	0	0	0	0
OTU248	Basidiomycota	Pucciniomycetes	Septobasidiales	Septobasidiaceae	Unidentified	Septobasidiaceae sp	0	0	43	0	0	1	0
OTU249	Basidiomycota	Cystobasidiomycetes	Erythrobasidiales	Unidentified	Unidentified	Cystobasidiales sp	354	0	1	0	0	0	1
OTU250	Basidiomycota	Cystobasidiomycetes	Erythrobasidiales	Symmetrosporaceae	Symmetrospora	Symmetrospora sp	0	0	4	0	2	0	0
OTU251	Basidiomycota	Pucciniomycetes	Septobasidiales	Septobasidiaceae	Unidentified	Septobasidiaceae sp	0	0	291	0	0	0	0
OTU252	Basidiomycota	Pucciniomycetes	Septobasidiales	Septobasidiaceae	Septobasidium	Septobasidium carestianum	0	0	0	0	0	5	1
OTU253	Basidiomycota	Pucciniomycetes	Septobasidiales	Septobasidiaceae	Septobasidium	Septobasidium velutinum	0	0	876	0	0	0	0
OTU254	Basidiomycota	Pucciniomycetes	Septobasidiales	Septobasidiaceae	Septobasidium	Septobasidium sinuosum	0	0	58	0	0	0	0
OTU255	Basidiomycota	Atractiellomycetes	Atractiellales	Helicogloeaceae	Helicogloea	Helicogloea dryina	0	0	0	12	0	0	0
OTU256	Basidiomycota	Tremellomycetes	Tremellales	Bulleraceae	Genolevuria	Genolevuria sp	0	0	1	0	0	0	6
OTU257	Basidiomycota	Pucciniomycetes	Septobasidiales	Septobasidiaceae	Unidentified	Unidentified	3	1	11	3	2	0	0
OTU258	Ascomycota	Lecanoromycetes	Lecanorales	Cladoniaceae	Cladonia	Cladonia sp	0	0	9	0	4	0	0
OTU259	Basidiomycota	Cystobasidiomycetes	Naohideales	Unidentified	Unidentified	Naohideales sp	0	0	0	4	0	0	1
OTU260	Ascomycota	Lecanoromycetes	Lecanorales	Cladoniaceae	Cladonia	Cladonia rei	15	1	0	4	35	13	1
OTU261	Unidentified	Unidentified	Unidentified	Unidentified	Unidentified	Fungi sp	0	0	1	0	0	0	0
OTU262	Unidentified	Unidentified	Unidentified	Unidentified	Unidentified	Fungi sp	1	3	0	14	0	0	0
OTU263	Basidiomycota	Tremellomycetes	Tremellales	Cuniculitremaceae	Kockovaella	Kockovaella sp	1	0	0	0	2	8	1
OTU264	Ascomycota	Lecanoromycetes	Lecanorales	Cladoniaceae	Cladonia	Cladonia rei	0	1	0	0	14	7	0

OTU265	Basidiomycota	Pucciniomycetes	Septobasidiales	Septobasidiaceae	Septobasidium	Septobasidium carestianum	0	0	0	0	0	6	0
OTU266	Basidiomycota	Tremellomycetes	Tremellales	Trimorphomycetaceae	Saitozyma	Saitozyma sp	1	0	0	0	0	0	0
OTU267	Basidiomycota	Tremellomycetes	Tremellales	Unidentified	Unidentified	Tremellales sp	0	0	2	0	0	0	0
OTU268	Basidiomycota	Agaricomycetes	Russulales	Lachnocladiaceae	Vararia	Vararia minidichophysa	0	0	0	5	0	0	0
OTU269	Basidiomycota	Cystobasidiomycetes	Erythrobasidiales	Erythrobasidiaceae	Erythrobasidium	Erythrobasidium sp	9	1	0	0	1	0	1
OTU270	Basidiomycota	Pucciniomycetes	Septobasidiales	Septobasidiaceae	Septobasidium	Septobasidium mariani	0	0	7	0	0	0	0
OTU271	Basidiomycota	Urediniomycetes	Urediniomycetes	Urediniomycetes	Curvibasidium	Curvibasidium pallidicorallinum	7	0	0	0	0	0	0
OTU272	Basidiomycota	Tremellomycetes	Tremellales	Bulleribasidiaceae	Hannaella	Hannaella sp	0	5	0	0	0	0	0
OTU273	Basidiomycota	Tremellomycetes	Tremellales	Cuniculitremaceae	Kockovaella	Kockovaella chinensis	0	0	3	0	0	0	0
OTU274	Unidentified	Unidentified	Unidentified	Unidentified	Unidentified	Fungi sp	0	0	3	0	0	0	0
OTU275	Basidiomycota	Cystobasidiomycetes	Erythrobasidiales	Unidentified	Unidentified	Erythrobasidiales sp	1	0	76	1	0	18	0
OTU276	Basidiomycota	Microbotryomycetes	Sporidiobolales	Sporidiobolaceae	Rhodotorula	Rhodotorula sp	34	0	0	0	0	0	3
OTU277	Basidiomycota	Agaricomycetes	Geastrales	Geastraceae	Geastrum	Geastrum triplex	0	0	0	1	3	0	0
OTU278	Basidiomycota	Microbotryomycetes	Sporidiobolales	Sporidiobolaceae	Sporobolomyces	Sporobolomyces sp	2	0	0	0	3	0	0
OTU279	Basidiomycota	Tremellomycetes	Tremellales	Tremellaceae	Tremella	Tremella diploschistina	2	6	11	13	6	1	0
OTU280	Basidiomycota	Microbotryomycetes	Microbotryomycetes	Heterogastridiaceae	Colacogloea	Colacogloea eucalyptica	0	0	6	0	0	0	0
OTU281	Basidiomycota	Agaricomycetes	Agaricales	Marasmiaceae	Marasmius	Marasmius sp	0	0	0	1	0	0	0
OTU282	Ascomycota	Lecanoromycetes	Lecanorales	Cladoniaceae	Cladonia	Cladonia rei	2	3	0	10	9	5	7
OTU283	Basidiomycota	Cystobasidiomycetes	Unidentified	Unidentified	Unidentified	Cystobasidiomycetes sp	0	0	0	0	0	0	4
OTU284	Basidiomycota	Tremellomycetes	Tremellales	Cuniculitremaceae	Kockovaella	Kockovaella sp	0	0	2	9	0	0	0
OTU285	Basidiomycota	Tremellomycetes	Tremellales	Cuniculitremaceae	Kockovaella	Kockovaella litseae	0	0	4	0	0	0	0
OTU286	Unidentified	Unidentified	Unidentified	Unidentified	Unidentified	Unidentified	1	0	0	8	0	0	0
OTU287	Basidiomycota	Pucciniomycetes	Septobasidiales	Septobasidiaceae	Septobasidium	Septobasidium sp	0	0	690	0	0	0	0
OTU288	Basidiomycota	Tremellomycetes	Tremellales	Cuniculitremaceae	Kockovaella	Kockovaella chinensis	0	0	6	0	0	0	0
OTU289	Unidentified	Unidentified	Unidentified	Unidentified	Unidentified	Fungi sp	0	0	0	10	0	0	0
OTU290	Basidiomycota	Tremellomycetes	Tremellales	Unidentified	Unidentified	Tremellales sp	0	0	0	0	6	0	0
OTU291	Basidiomycota	Cystobasidiomycetes	Unidentified	Unidentified	Unidentified	Unidentified	1	3	1	0	0	0	0
OTU292	Basidiomycota	Microbotryomycetes	Microbotryomycetes	Chrysozymaceae	Oberwinklerozyma	Oberwinklerozyma silvestris	0	0	9	0	0	0	0
OTU293	Unidentified	Unidentified	Unidentified	Unidentified	Unidentified	Fungi sp	0	0	16	0	0	0	0
OTU294	Basidiomycota	Cystobasidiomycetes	Erythrobasidiales	Erythrobasidiaceae	Erythrobasidium	Erythrobasidium sp	2	0	0	0	0	0	0
OTU295	Basidiomycota	Tremellomycetes	Tremellales	Unidentified	Unidentified	Tremellales sp	0	0	5	0	1	0	0

OTU296	Basidiomycota	Cystobasidiomycetes	Erythrobasidiales	Microsporomycetaceae	Unidentified	Microsporomycetaceae sp	2	0	0	0	0	0	0
OTU297	Ascomycota	Lecanoromycetes	Lecanorales	Cladoniaceae	Cladonia	Cladonia sp	0	21	66	5	18	1	0
OTU298	Ascomycota	Lecanoromycetes	Lecanorales	Cladoniaceae	Cladonia	Cladonia rei	49	6	0	20	33	0	12
OTU299	Basidiomycota	Tremellomycetes	Tremellales	Cuniculitremaceae	Kockovaella	Kockovaella sp	0	0	0	3	0	0	0
OTU300	Basidiomycota	Agaricomycetes	Hymenochaetales	Schizoporaceae	Hyphodontia	Hyphodontia sp	0	0	0	0	0	0	2
OTU301	Basidiomycota	Cystobasidiomycetes	Cystobasidiales	Cystobasidiaceae	Occultifur	Occultifur mephitis	15	0	0	0	0	0	0
OTU302	Basidiomycota	Cystobasidiomycetes	Erythrobasidiales	Unidentified	Unidentified	Erythrobasidiales sp	4	0	0	0	0	0	0
OTU303	Ascomycota	Lecanoromycetes	Lecanorales	Cladoniaceae	Cladonia	Cladonia rei	0	0	0	0	0	110	2
OTU304	Basidiomycota	Pucciniomycetes	Septobasidiales	Septobasidiaceae	Septobasidium	Septobasidium carestianum	0	0	0	0	0	3	0
OTU305	Basidiomycota	Tremellomycetes	Tremellales	Bulleribasidiaceae	Hannaella	Hannaella sp	0	0	0	2	0	0	0
OTU306	Basidiomycota	Pucciniomycetes	Septobasidiales	Septobasidiaceae	Septobasidium	Septobasidium carestianum	0	0	1	3	0	11	0
OTU307	Basidiomycota	Pucciniomycetes	Septobasidiales	Septobasidiaceae	Septobasidium	Septobasidium sp	0	0	0	0	0	9	0
OTU308	Ascomycota	Lecanoromycetes	Lecanorales	Cladoniaceae	Cladonia	Cladonia rei	13	6	20	0	4	0	0
OTU309	Unidentified	Unidentified	Unidentified	Unidentified	Unidentified	Unidentified	2	1	2	0	0	11	0
OTU310	Basidiomycota	Cystobasidiomycetes	Erythrobasidiales	Erythrobasidiaceae	Bannoa	Bannoa ogasawarensis	0	0	0	102	0	0	0
OTU311	Basidiomycota	Microbotryomycetes	Sporidiobolales	Sporidiobolaceae	Rhodotorula	Rhodotorula mucilaginosa	0	2	0	0	1	0	0
OTU312	Basidiomycota	Cystobasidiomycetes	Cystobasidiales	Cystobasidiaceae	Cystobasidium	Cystobasidium sp	0	0	0	0	0	14	0
OTU313	Ascomycota	Lecanoromycetes	Lecanorales	Cladoniaceae	Cladonia	Cladonia sp	0	3	1	29	4	0	8
OTU314	Basidiomycota	Pucciniomycetes	Septobasidiales	Septobasidiaceae	Septobasidium	Septobasidium pallidum	0	0	0	0	0	6	0
OTU315	Basidiomycota	Pucciniomycetes	Septobasidiales	Septobasidiaceae	Septobasidium	Septobasidium carestianum	0	0	0	0	0	11	0
OTU316	Basidiomycota	Tremellomycetes	Tremellales	Trimorphomycetaceae	Carlosrosaea	Carlosrosaea sp	0	0	3	0	0	0	0
OTU317	Basidiomycota	Pucciniomycetes	Septobasidiales	Septobasidiaceae	Unidentified	Septobasidiaceae sp	0	0	5	0	0	0	0
OTU318	Basidiomycota	Pucciniomycetes	Septobasidiales	Septobasidiaceae	Septobasidium	Septobasidium mariani	0	0	3	0	0	0	0
OTU319	Basidiomycota	Pucciniomycetes	Septobasidiales	Septobasidiaceae	Septobasidium	Septobasidium carestianum	0	0	0	0	0	2	0
OTU320	Basidiomycota	Agaricomycetes	Russulales	Wrightoporiaceae	Wrightoporia	Wrightoporia sp	0	0	4	0	0	0	0
OTU321	Basidiomycota	Cystobasidiomycetes	Cystobasidiales	Cystobasidiaceae	Cystobasidium	Cystobasidium sp	0	0	23	1	0	0	0
OTU322	Basidiomycota	Cystobasidiomycetes	Cystobasidiales	Cystobasidiaceae	Occultifur	Occultifur mephitis	6	0	0	0	0	0	0
OTU323	Basidiomycota	Unidentified	Unidentified	Unidentified	Unidentified	GS27 sp	0	0	6	1	0	0	0
OTU324	Basidiomycota	Agaricomycetes	Corticiales	Corticiaceae	Corticium	Corticium sp	0	0	2	0	0	0	0
OTU325	Basidiomycota	Agaricomycetes	Agaricales	Lycoperdaceae	Lycoperdon	Lycoperdon perlatum	0	0	0	0	2	1	0

OTU326	Basidiomycota	Tremellomycetes	Tremellales	Bulleribasidiaceae	Hannaella	Hannaella kunmingensis	0	0	0	0	2	5	0
OTU327	Basidiomycota	Cystobasidiomycetes	Erythrobasidiales	Erythrobasidiaceae	Erythrobasidium	Erythrobasidium sp	3	0	0	0	0	0	0
OTU328	Basidiomycota	Pucciniomycetes	Septobasidiales	Septobasidiaceae	Septobasidium	Septobasidium carestianum	0	0	0	0	0	5	0
OTU329	Basidiomycota	Microbotryomycetes	Microbotryomycetes	Heterogastridiaceae	Colacogloea	Colacogloea sp	19	0	0	0	0	0	2
OTU330	Basidiomycota	Agaricomycetes	Boletales	Suillaceae	Suillus	Suillus sp	0	14	0	0	0	0	0
OTU331	Basidiomycota	Tremellomycetes	Tremellales	Unidentified	Unidentified	Tremellales sp	3	0	0	0	0	0	0
OTU332	Basidiomycota	Pucciniomycetes	Septobasidiales	Septobasidiaceae	Unidentified	Septobasidiaceae sp	0	0	17	0	0	1	0
OTU333	Ascomycota	Lecanoromycetes	Lecanorales	Cladoniaceae	Cladonia	Cladonia sp	0	0	20	0	0	0	0
OTU334	Basidiomycota	Agaricomycetes	Hymenochaetales	Schizoporaceae	Hyphodontia	Hyphodontia rhizomorpha	0	0	0	0	0	2	0
OTU335	Unidentified	Unidentified	Unidentified	Unidentified	Unidentified	Unidentified	1	2	0	0	0	0	6
OTU336	Unidentified	Unidentified	Unidentified	Unidentified	Unidentified	Fungi sp	9	0	0	0	0	0	0
OTU337	Basidiomycota	Cystobasidiomycetes	Erythrobasidiales	Unidentified	Unidentified	Unidentified	0	0	3	0	4	0	
OTU338	Basidiomycota	Cystobasidiomycetes	Erythrobasidiales	Erythrobasidiaceae	Bannoa	Bannoa ogasawarensis	16	0	0	2	14	0	0
OTU339	Unidentified	Unidentified	Unidentified	Unidentified	Unidentified	Unidentified	0	0	0	0	0	22	0
OTU340	Basidiomycota	Cystobasidiomycetes	Cystobasidiales	Cystobasidiaceae	Occultifur	Occultifur mephitis	3	0	0	0	0	0	0
OTU341	Basidiomycota	Cystobasidiomycetes	Cystobasidiales	Cystobasidiaceae	Occultifur	Occultifur sp	1	0	0	0	0	0	0
OTU342	Basidiomycota	Cystobasidiomycetes	Cystobasidiales	Cystobasidiaceae	Cystobasidium	Cystobasidium sp	0	0	1	4	0	0	1
OTU343	Basidiomycota	Cystobasidiomycetes	Cystobasidiales	Cystobasidiaceae	Cystobasidium	Cystobasidium sp	3	0	0	0	0	0	0
OTU344	Basidiomycota	Agaricomycetes	Amylocorticiales	Amylocorticiaceae	Amyloathelia	Amyloathelia crassiuscula	0	0	2	0	0	2	0
OTU345	Basidiomycota	Microbotryomycetes	Microbotryomycetes	Chrysozymaceae	Bannozyma	Bannozyma yamatoana	0	0	1	1	0	41	0
OTU346	Ascomycota	Lecanoromycetes	Lecanorales	Cladoniaceae	Cladonia	Cladonia rei	0	0	2	1	2	4	0
OTU347	Basidiomycota	Microbotryomycetes	Microbotryomycetes	Heterogastridiaceae	Colacogloea	Colacogloea sp	0	0	0	1	4	0	0
OTU348	Basidiomycota	Cystobasidiomycetes	Erythrobasidiales	Unidentified	Unidentified	Erythrobasidiales sp	0	0	7	0	0	0	0

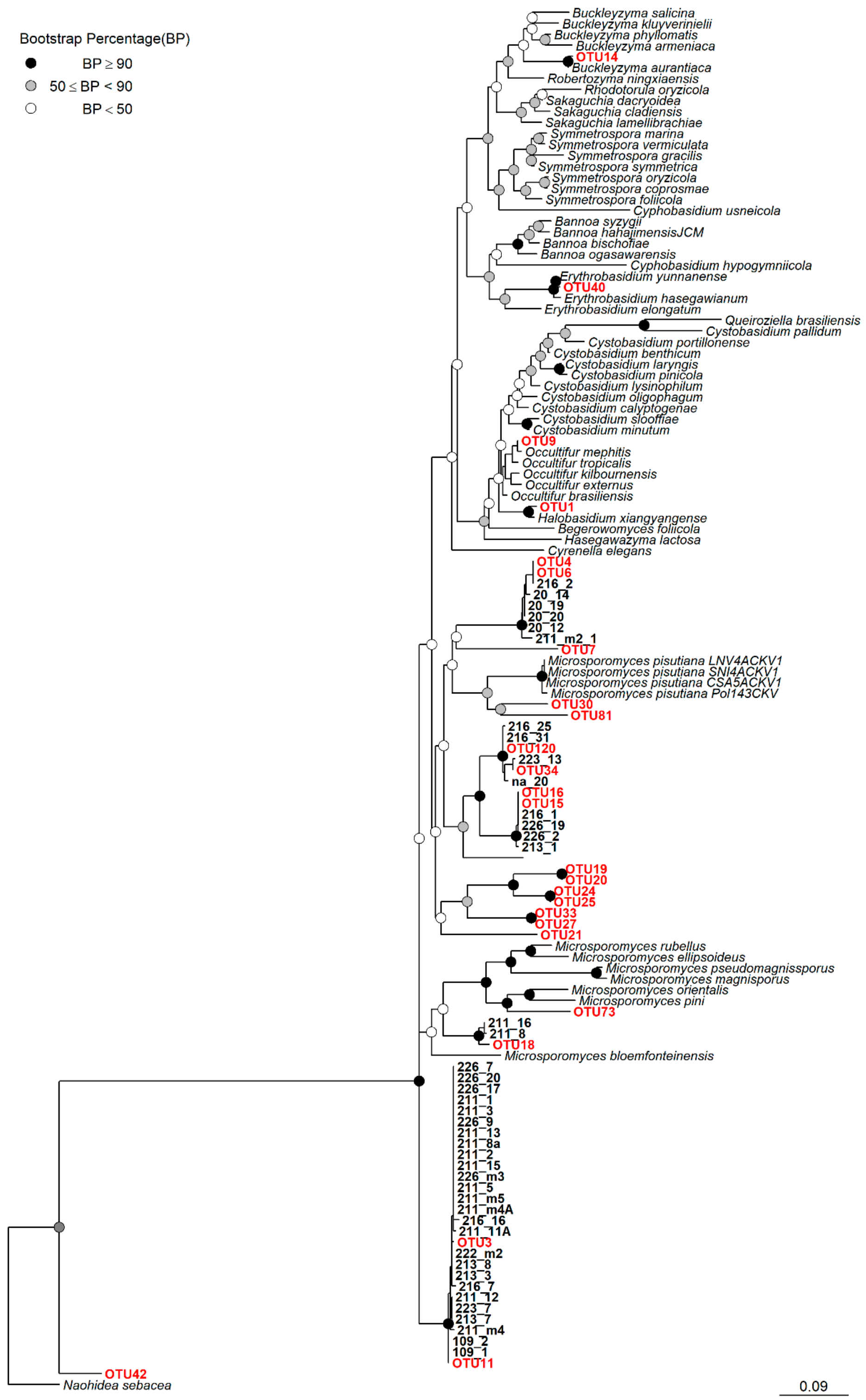

Figure S1. Phylogeny tree of *Cystobasidiomycetes* obtained via the maximum likelihood analysis of the ITS sequences. The OTUs from the meta-barcoding sequences were marked with red color.

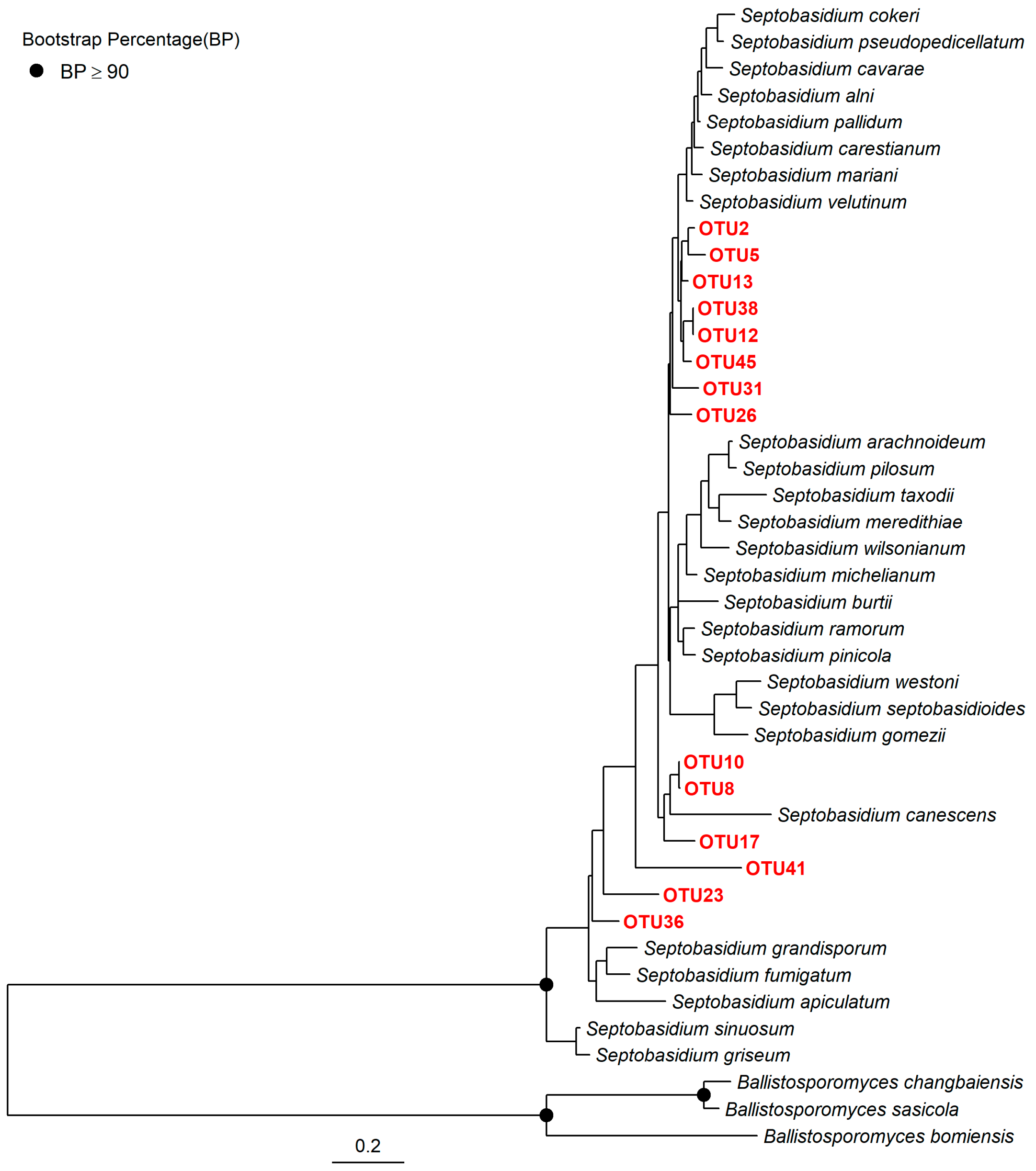

Figure S2. Phylogeny tree of *Septobasidium* obtained by maximum likelihood analysis of the ITS sequences. The OTUs from the meta-barcoding sequences were marked with red color.
